# Maximizing efficiency in sunflower breeding through historical data optimization

**DOI:** 10.1186/s13007-024-01151-0

**Published:** 2024-03-16

**Authors:** Javier Fernández-González, Bertrand Haquin, Eliette Combes, Karine Bernard, Alix Allard, Julio Isidro y Sánchez

**Affiliations:** 1 Centro de Biotecnologia y Genómica de Plantas (CBGP, UPM-INIA)—Instituto Nacional de Investigación y Tecnologia Agraria y Alimentaria (INIA), Universidad Politécnica de Madrid (UPM), Campus de Montegancedo-UPM, Pozuelo de Alarcón, Madrid 28223 Spain; 2 Syngenta, Saint-Sauveur, France

**Keywords:** Genomic selection, Training set optimization, Sunflower hybrids, Historical data, Multi-objective optimization

## Abstract

Genomic selection (GS) has become an increasingly popular tool in plant breeding programs, propelled by declining genotyping costs, an increase in computational power, and rediscovery of the best linear unbiased prediction methodology over the past two decades. This development has led to an accumulation of extensive historical datasets with genotypic and phenotypic information, triggering the question of how to best utilize these datasets. Here, we investigate whether all available data or a subset should be used to calibrate GS models for across-year predictions in a 7-year dataset of a commercial hybrid sunflower breeding program. We employed a multi-objective optimization approach to determine the ideal years to include in the training set (TRS). Next, for a given combination of TRS years, we further optimized the TRS size and its genetic composition. We developed the Min_GRM size optimization method which consistently found the optimal TRS size, reducing dimensionality by 20% with an approximately 1% loss in predictive ability. Additionally, the Tails_GEGVs algorithm displayed potential, outperforming the use of all data by using just 60% of it for grain yield, a high-complexity, low-heritability trait. Moreover, maximizing the genetic diversity of the TRS resulted in a consistent predictive ability across the entire range of genotypic values in the test set. Interestingly, the Tails_GEGVs algorithm, due to its ability to leverage heterogeneity, enhanced predictive performance for key hybrids with extreme genotypic values. Our study provides new insights into the optimal utilization of historical data in plant breeding programs, resulting in improved GS model predictive ability.

**Supplementary Information:**

The online version contains supplementary material available at 10.1186/s13007-024-01151-0.

## Background

Sunflower (*Helianthus annuus L.*) is a globally significant crop, being the fourth largest source of vegetable oil and the second most important hybrid crop [1]. Initially, traditional sunflower breeding relied on open-pollinated varieties. However, the discovery of cytoplasmic male sterility and fertility restoration genes brought about a shift towards hybrid breeding, resulting in improved yield and genotypic uniformity of cultivars [2, 3]. Although marker-assisted selection (MAS) has been used in sunflower breeding to select for specific traits such as disease resistance, herbicide tolerance, and fertility restoration genes [1, 4], it is not suitable for complex traits like yield and oil content [5–7]. However, genomic technologies have transformed breeding by enabling genomic selection (GS) [8], which plays a crucial role in identifying and selecting plants with desirable quantitative traits [9, 10]. Genomic selection has been implemented in both self [11–13] and cross-pollinated [14] species. In sunflower breeding, GS has revolutionized the process, providing a more efficient and effective means of improving crop yield and quality. Recently, Livaja et al. [15] developed a 25k SNP array from a wide variety of sunflower germplasm. This provides a valuable resource for implementating GS in sunflower research. This array was validated using genomic predictions for *Sclerotinia* resistance, although GS is especially well suited for more quantitative traits such as yield and oil content. In this context, GS has been shown to outperform classical general combining ability (GCA) approaches. This is especially true when predicting hybrids with poorly characterized parents [16, 17].

Constructing a statistical GS model requires a training set (TRS) that includes genotyped and phenotyped individuals. The effectiveness of GS is heavily reliant on the quality of the TRS used, as demonstrated by several studies [18]. To ensure maximum efficiency, it is essential to optimize the TRS, with the goal of maximizing both genetic diversity and the relationship between the TRS and the test set (TS) whose genomic estimated genotypic values (GEGVs) are to be predicted [18–21]. TRS optimization typically  involves  selecting a smaller TRS as a subset of a larger candidate set. This can be accomplished through either targeted or untargeted methods [18, 22]. The former requires knowledge of the genotypes of the TS during optimization, leading to a substantial increase in performance, while the latter increases diversity without information about the TS [22]. The size of the TRS is also a critical factor in optimizing GS, and should be maximized for the best results. However, beyond a certain point, further increasing its size becomes costly and leads to diminishing returns [19, 22–28].

TRS optimization of historical data offers two potential benefits: (i) enhancing prediction accuracy by removing hybrids weakly related to the TS and (ii) reducing data dimensionality, streamlining data management and computational efficiency in subsequent analyses [10, 18, 24]. Our primary objective is to enhance predictive GS models, acknowledging their  pivotal role in influencing the efficacy of selection responses. While not typically a limiting factor,  the extensive data dimensions typical in commercial breeding programs can slow GS models training times. Previous studies on TRS optimization have mainly addressed within-year and within-generation scenarios [21, 22, 24, 26, 27, 29–41] for self-pollinated and hybrid crops using cross-validation. Studies have also investigated genomic predictions across years for hybrids without optimization, using both simulations [42, 43] and empirical approaches [44–46]. However, research on optimization across years and generations for the efficient use of historical data in hybrid crops is lacking. Despite numerous studies, this specific scenario remains unexplored. Although Neyhart et al. [47] used TRS optimization algorithms for long-term recurrent selection in barley, a self-pollinated crop, they did not focus on optimizing historical data. Similarly, Tayeh et al. [48] applied TRS optimization for across-year predictions in peas,  yet this study differs from our scenario in key aspects. Firstly, the crop studied was self-pollinated, optimization was only applied within generation, small population sizes were considered, and only the mean of the coefficient of determination (CDmean) algorithm was tested. Fernandez-González et al. [19] showed that, while this algorithm is powerful, its slow performance poses  challenges for application to the large-scale datasets commonly encountered in industry.

Although many studies have explored TRS optimization, most have tested various TRS sizes without proposing a systematic method to identify the optimal size a priori. While this approach is reasonable when dealing with sparse testing, in which TRS size can be determined by the limited available resources for field phenotyping, it is crucial to optimize both the TRS size and composition when working with historical data. Recent literature, such as studies conducted by Fernández-González et al. [19] and Wu et al. [28], suggest algorithms for systematically determining the optimal size of a TRS. Yet, the implications of integrating historical data into the TRS for its predictive performance remains an under-explored area of research. To address this gap, we focused on the role of historical genotypic and phenotypic data from a commercial sunflower breeding program in optimizing the TRS’s size and composition.

## Results

### Population structure

**Fig. 1:**
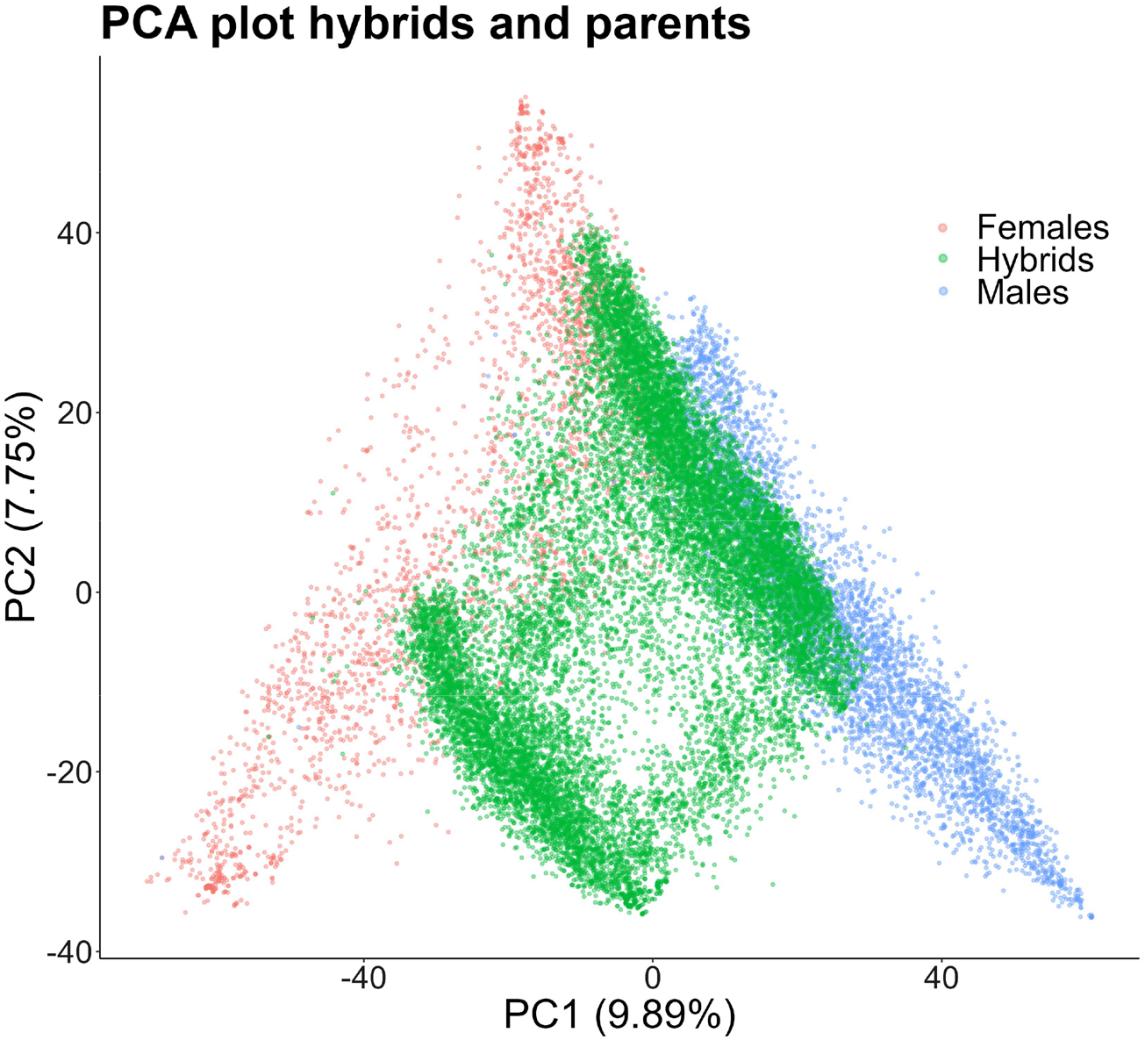
This plot displays the first two principal components, calculated on genome-wide marker data, which explained 17 % of the genetic variance in the population studied. Each solid circle represents a genotype, with colors indicating membership to male parental lines, female parental lines, or their hybrid combinations

We analyzed the genetic relationships between parental lines and hybrids of sunflower within a multivariate genetic space, defined by the genome-wide markers for each genotype in our dataset. This complex space can be summarized by the first two principal components in a principal components analyses (PCA) of the marker data,  allowing to visualize it in two dimensions as illustrated in Fig. 1., We found that male and female groups overlap in the higher values of PC2 but diverge with decreasing PC2 values. Hybrids are positioned between the parental groups, exhibiting varying overlap with them. This overlap is specially pronounced with the male group and in the upper portion of the plot. Most hybrids can be grouped in two clusters separated along the axis followed by female parental lines. As the parental populations exhibited no substantial population structure, it was unnecessary to incorporate clustering information into the optimization of the training set.

### Optimization of the years to be included into the training set

**Table 1:** Predictive ability of GBLUP and GBM models across all traits using all available data for the selected years to calibrate them

Trait	Model	Test Set	Oldest year included in the training set								
			6	5	4	3	2	1		2*	1*
YLD	GBM	6	NA	0.344	0.370	*0.352*	0.356	0.356		**0.384**	*0.383*
		7	0.328	0.358	0.374	*0.372*	**0.384**	*0.383*		0.381	*0.377*
	GBLUP	6	NA	0.368	*0.365*	*0.337*	0.344	0.357		**0.370**	0.380
		7	0.338	0.369	0.374	*0.368*	**0.377**	*0.373*		0.379	*0.374*
GM	GBM	6	NA	0.418	0.446	0.460	**0.463**	0.475		*0.445*	0.453
		7	0.432	0.467	0.481	*0.473*	**0.475**	0.486		0.487	0.502
	GBLUP	6	NA	0.390	0.398	0.420	**0.425**	0.427		0.401	0.407
		7	0.400	0.436	0.481	0.489	***0.480***	0.490		*0.475*	0.488
OIL	GBM	6	NA	0.467	0.531	0.548	**0.557**	*0.556*		0.549	0.550
		7	0.419	0.424	0.464	0.467	**0.470**	0.478		0.467	0.471
	GBLUP	6	NA	0.512	0.542	*0.539*	**0.546**	0.551		0.555	0.560
		7	0.420	0.461	0.488	0.489	**0.493**	*0.488*		0.495	*0.488*

The analysis considered various training set-test set combinations, with two test sets representing data for years 6 and 7. The training sets were constructed by including data from the year preceding the test set, two years prior to the test set, and so on until all available data older than the test set was included. We have highlighted in italic all cases where adding an additional year resulted in reduced predictive ability and in bold the year combinations selected by multi-objective optimization. The training sets with an asterisk next to them (Last two columns) indicate that year 3 has been excluded from them. Although these latter two scenarios were not among those initially planned, in light of the results from the multi-objective optimization, we decided to include them in our analysis

We conducted an analysis of eleven different scenarios summarized in Table 5 to investigate whether including older data in the TRS would enhance or reduce predictive ability. The results presented in Table 1 revealed that predictive ability ranged from 0.328 to 0.384 in YLD, 0.400 to 0.490 in GM and 0.419 to 0.560 in OIL. Our results showed that the predictive ability generally improved as we increased the number of years in the TRS. However, we noticed diminishing returns when adding more years. Specifically, the inclusion of a second year in the TRS led to an average increase of 6.84% in predictive ability, while the addition of the oldest year resulted in a smaller average increase of 0.93%. However, we identified several exceptions to this general trend, which are highlighted in italic in Table 1. For instance, while including the oldest year (year 1) generally improved model performance, it had the opposite effect in 4 out of 12 cases (third last column in Table 1), with its impact on predictive ability ranging from a reduction of 1.06% to an increase of 3.78%. We also found that including year 4 in the analysis led to a decrease in predictive ability for YLD by 0.82% when year 6 was the test set. Similarly, when we included year 2 in the GBLUP analysis with year 7 as the test set for GM, we noticed a reduction in predictive ability by 1.84%. These two instances, marked by their reductions rather than improvements, can be seen as outliers in our generally observed trend of enhanced performance. Finally, we found that year 3 had the most consistent negative effect on the predictive ability of the models. In particular, for YLD, including year 3 in the TRS caused a reduction of predictive ability ranging from 0.53% to 7.67%, depending on the TS and model used. In contrast, in GM and OIL, it improved performance in 3 out of 4 scenarios and decreased it in the remaining scenario. Excluding year 3 from a TRS that contains the older years 1 or 2 (last two columns in Table 1), resulted in a strong increase in predictive ability for YLD, a substantial reduction in most GM scenarios and minor changes in OIL.

**Fig. 2:**
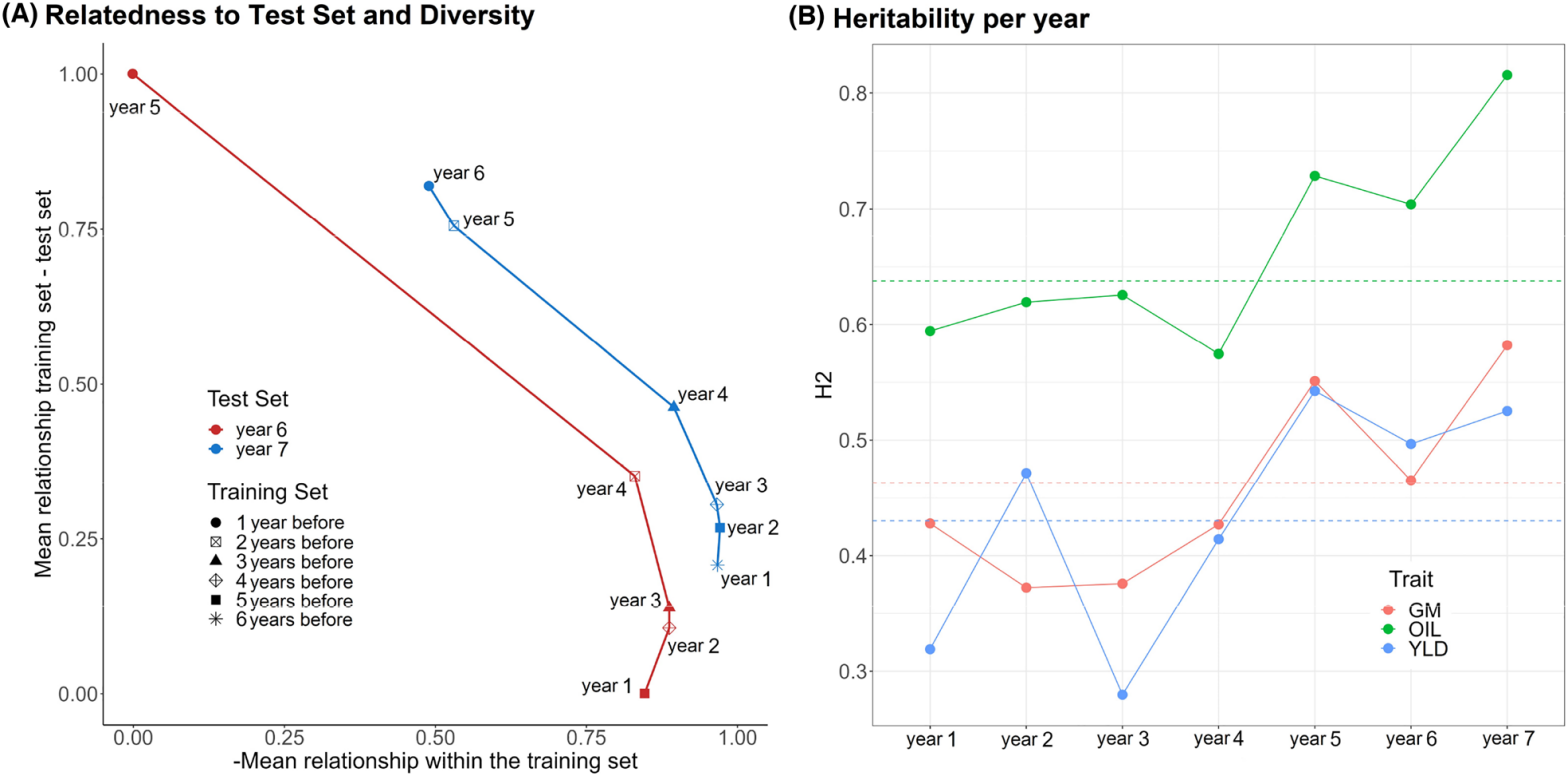
**A** Trade-off between the average additive relationship between the training and test sets (vertical axis) and the opposite value of the average additive relationship within the training set, i.e. training set diversity (horizontal axis). The values in both axes have been normalized between 0 and 1. Each point corresponds to one of the eleven combinations between training and test set years tested in this work. The point shape refers to the number of consecutive years preceding the test set that we used to build the training population, with labels indicating the oldest year contained in the training set. Within the training sets, all available data for the corresponding years has been considered (no optimization). **B** Broad sense heritability for each trait within each year. The horizontal dashed lines correspond to the heritability calculated across all years

We elaborated Fig. 2 to interpret the results in Table 1. In Fig. 2A, a trade-off between the relationship to the TS and diversity can be observed. As we progressively included older years into the TRS, we noted a consistent decrease in its average relationship with the TS and an increase in diversity. The diversity gain was rapid initially, but it slowed down for the inclusion of years 3 and 2, and adding year 1 slightly reduced average diversity. For all traits in our study, we observed that years 5, 6, and 7 demonstrated higher heritability compared to the older years, as illustrated in Fig. 2B. It is important to remark that YLD presented a strong drop in heritability for years 1 and 3, which match the reductions in predictive ability observed in Table 1.

**Fig. 3:**
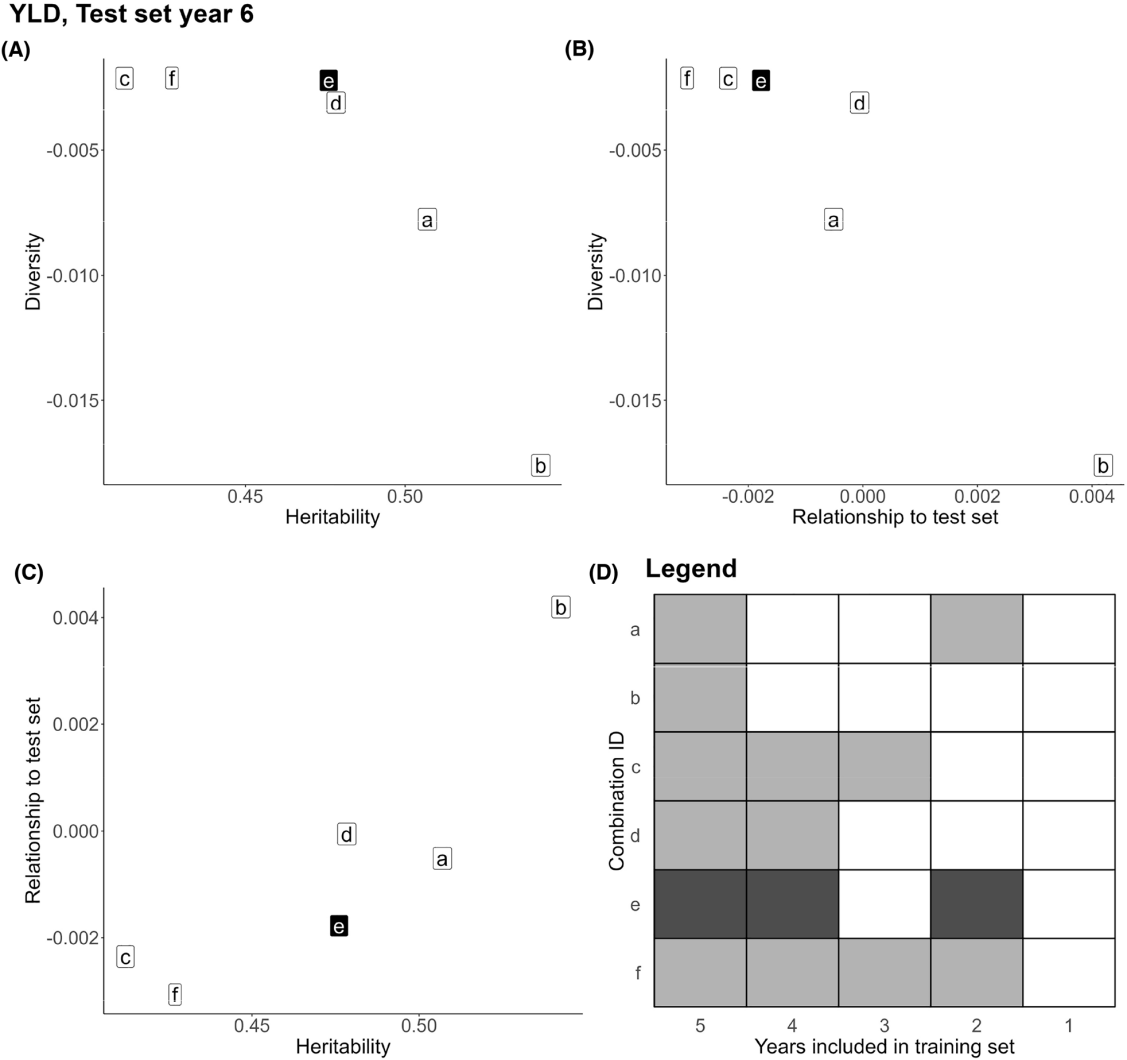
Results of the multi-objective optimization that aimed to maximize diversity, relationship to the test set, and average heritability in the yield trait when the test set was year 6. The solutions obtained from the optimization algorithm form a three-dimensional Pareto front. For ease of result visualization, the findings are presented in three two-dimensional plots, showcasing pairwise combinations of the variables maximized during the optimization: **A** Diversity against heritability. **B** Diversity against relationship to the test set. **C** Relationship to the test set against heritability. In these plots, each letter represents a year combination from the Pareto front, and the composition of each combination is shown in plot (**D**). Gray squares indicate the years included in the training set, while a darker-colored square highlights the year combination corresponding to the best solution (*e*)

With the aim of finding the optimal TRS years, we leveraged the trade-off between the relationship to the TS, diversity, and heritability through multi-objective optimization. Our aim was to maximize these three variables simultaneously, as shown in Figs. 3, Additional file 3: Figs. S4 to S8. The results revealed a clear trade-off between diversity and the other two variables, whereby an increase in one variable led to a decrease in the other (Fig. 3A and B). Conversely, there was a positive relationship between relationship to the TS and heritability (Fig. 3 C). Year combinations with high heritability and relationship to the TS, such as solution *b* in Fig. 3, exhibited lower diversity due to the inclusion of fewer years in the TRS. However, these combinations did not perform as well as others (Table 1). Solutions with extremely high diversity (solutions *c, f, e*) achieved very high predictive abilities. Among them, solution *e* maximized both heritability and relationship to the TS (Fig. 3A–C). This solution corresponded to years 2, 4, and 5 (Fig. 3 D) and yielded the best predictive ability for GBM and the second best for GBLUP (Table 1). For further details on other traits and TS years, please refer to the Additional file 3, Note 7. The optimal year combinations selected for each trait are highlighted in bold in Table 1.

Through our analysis, we discovered a consistent approach for identifying the best-performing solutions among those suggested by the multi-objective optimization for all traits and TS years. This approach involved two steps: (i) Selecting solutions with extremely high diversity and discarding the remaining options (Figs. 3 and Additional file 3: Figs. S4−S8; A, B). (ii) Among the solutions with the highest diversity, selecting the one that maximizes the number of years included in the TRS (Figs. 3 and Additional file 3: Fig. S4–S8; D), as well as heritability and relationship to the TS (Figs. 3 and Additional file 3: Fig. S4–S8; A, B, C). By following this methodology, we consistently identified combinations of years for the TRS that exhibited the highest performance, as shown in Table 1. The optimized solutions were either the best or extremely close to the best for YLD and OIL traits, while their predictive ability for GM ranged between 94.6% and 99.5% of the highest achieved value.

### Optimization of training set composition for fixed training set sizes

**Fig. 4:**
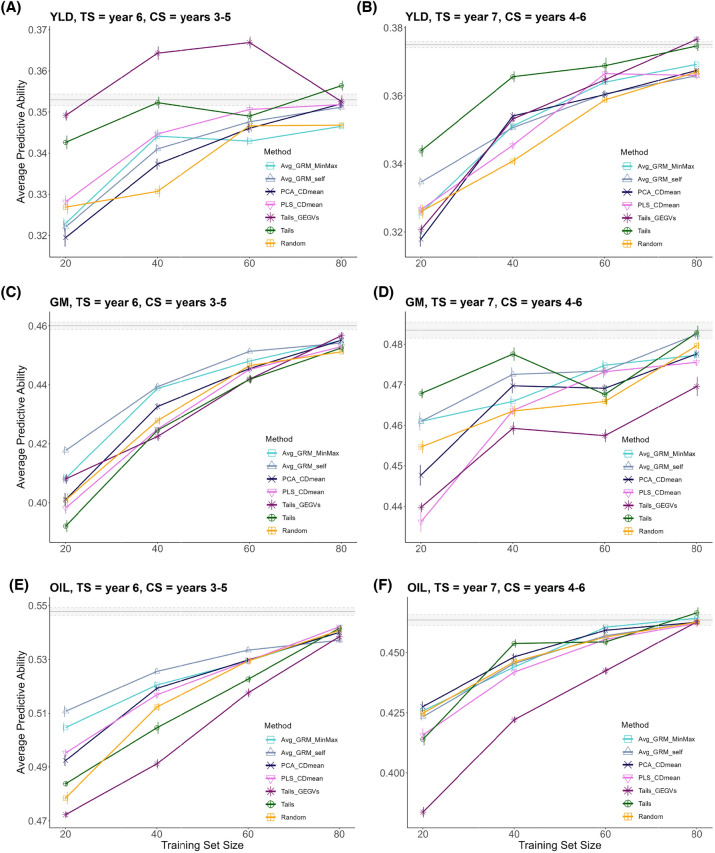
Relationship between training set size and predictive ability of models for grain yield (YLD), grain moisture (GM), and percentage of oil (OIL), calibrated with TRS obtained by various optimization methods. The plot shows the average predictive ability across iterations of the training set optimization and repetitions of the gradient boosting machine model for two different combinations of candidate and test set years. The x-axis represents the size of the training set as a percentage of the candidate set. Error bars indicate the standard error of the mean. The gray horizontal line represents the average predictive ability achieved when using the entire candidate set to calibrate the prediction models and the gray area around it shows the standard error of the mean

After selecting the years to be included in the TRS (Fig. 3), the genetic composition can be further optimized using different optimization methods. We comprehensively evaluated the predictive ability of optimization methods for YLD, GM, and OIL using various combinations of years in the candidate set and TS (Table 5). Figure 4 presents the evolution of the predictive ability for all methods as the TRS size increases in two scenarios (TS year 6, CS years 3–5 and TS year 7, CS years 4–6) that showcase the general trends found in the eleven scenarios tested (Table 5). Detailed results for all scenarios and repetitions are available in the Additional files 1, 2. Our analysis showed that the predictive ability generally improved as the TRS size increases. However, the rate of improvement diminishes for larger TRS sizes. Additionally, we observe that the difference in predictive ability between optimization methods is more prominent for small TRS sizes than for larger ones.

Tails_GEGVs for YLD demonstrated very distinctive performance trends, achieving maximum performance for intermediate TRS sizes and declining for larger sizes (Fig. 4A). This trend occurred in 54.5% of the scenarios tested (Table 5), with maximum predictive ability typically occurring at a TRS size of 60% of the candidate set. Tails_GEGVs outperformed using the entire candidate set to calibrate the models in 54.5% of the scenarios for YLD; 36.4% for OIL and 27.3% for GM, although the TRS size at which it occurred was not consistent. The Tails method outperformed the use of the entire candidate set as frequently as Tails_GEGVs did. Other methods, in contrast, did not achieve this level of performance, although they often managed a slightly lower or similar predictive ability than using all data with a TRS size equating to 80% of the candidate set.

Table 2 provides a more detailed overview of the relative performance of optimization methods across TRS sizes. We found that optimization methods performed best in YLD, with an average area under the curve (AUC) gain of 1.66% across methods and scenarios, followed by GM (0.12% AUC gain) and OIL (– 0.12%).

Among the optimization methods, Tails and Tails_GEGVs had the best average performance in YLD, with AUC gains of 4.16% and 2.93%, respectively. As shown in Fig. 4 B, Tails_GEGVs usually reached a higher maximum accuracy than Tails, but Tails had a better performance across the entire range of sizes, resulting in a larger AUC value. However, for the other traits, Tails and Tails_GEGVs had poor performance and were typically worse than random sampling (negative AUC gain). Genetic-based methods, such as Avg_GRM_self and Avg_GRM_MinMax, showed much higher consistency across traits, with average AUC gains across scenarios ranging from 0.75 to 1.16% depending on the trait. Average AUC gain for PCA_CDmean ranged from 0.11 to 0.6%. PLS_CDmean, which includes both phenotypic and genotypic information, generally performed similarly to PCA_CDmean but with a larger variance, with average AUC gains ranging from − 0.41 to 0.7%.

We evaluated the consistency of the methods within each trait by calculating the variance across scenarios (penultimate column in Table 2). YLD had the highest variances, with all methods ranging between 1.5 and 3 except Tails_GEGVs, which had a variance of 8.31. The high variance of Tails_GEGVs is due to the fact that this method performed substantially better for TS year 6 (4.94 AUC gain on average) than for TS year 7 (1.26 average AUC gain). For GM and OIL, the variances were notably lower, ranging between 0.5 and 1.5 for all methods except Tails and Tails_GEGVs, which ranged between 7 and 12.

**Table 2:** Overview of the average performance of the optimization methods in all traits and scenarios measured as the percentage of gain in area under the curve (AUC) relative to random sampling

Optimization type	Optimization methods	Test set year 6						Test set year 7							Global average	Variance	Trait
		CS year 5	CS year 4-5	CS year 3–5	CS year 2–5	CS year 1–5	Test set average	CS year 6	CS year 5–6	CS year 4-6	CS year 3–6	CS year 2–6	CS year 1–6	Test set average			
Genetic-based	Avg_GRM_self	− 1.17	2.89	1.11	− 0.94	− 0.11	0.36	2.25	3.46	1.51	0.13	− 0.5	0.47	1.22	0.83	2.41	YLD
	Avg_GRM_MinMax	0.37	2.35	0.78	0.12	0.64	0.85	0.54	3.64	1.6	− 0.44	− 0.58	0.8	0.93	0.89	1.52	
	PCA_CDmean	0.25	1.94	0.51	1.43	1.38	1.1	0.27	2.54	1.07	− 1.96	− 0.93	− 1.43	− 0.07	0.46	2.01	
Mixed	PLS_CDmean	0.6	1.86	2.1	0.01	1.8	1.27	− 0.06	3.41	1.21	− 1.63	− 1.33	− 0.25	0.23	0.7	2.36	
	Tails_GEGVs	0.3	4.83	7.66	4.55	7.35	4.94	2.22	3.02	1.48	− 1.35	1.55	0.66	1.26	2.93	8.31	
Phenotypic-based	Tails	0.53	5.98	3.72	2.38	4.07	3.34	4.93	6.38	5.97	3.99	3.93	3.83	4.84	4.16	2.88	
	Scenario average	0.15	3.31	2.65	1.26	2.52	1.98	1.69	3.74	2.14	− 0.21	0.36	0.68	1.4	1.66	1.76	
	Test set average			1.98						1.4					1.66	0.09	
Genetic-based	Avg_GRM_self	1.18	1.85	2.06	0.41	− 0.34	1.03	0.05	− 0.38	1.53	0.53	2.31	1.48	0.92	0.97	0.93	GM
	Avg_GRM_MinMax	1.52	2.18	1.43	0.55	0.65	1.27	1.08	− 0.05	0.98	1.18	2.17	1.08	1.07	1.16	0.44	
	PCA_CDmean	− 0.74	0.43	0.48	− 0.42	− 0.63	− 0.18	− 0.57	0.52	0.37	− 0.13	1.62	0.24	0.34	0.11	0.48	
Mixed	PLS_CDmean	0.03	− 0.06	− 0.33	− 0.43	− 1.27	− 0.41	− 1.28	− 1.37	− 0.25	− 0.74	0.18	0.98	− 0.41	− 0.41	0.52	
	Tails_GEGVs	− 3.82	0.2	− 1.19	0.71	1.09	− 0.6	2.49	1.69	− 2.22	− 3.16	− 5.06	− 4.58	− 1.81	− 1.26	7.09	
Phenotypic-based	Tails	− 8.5	− 0.12	− 1.31	0.06	0.92	− 1.79	3.8	2.11	1.54	1.73	0.89	0.43	1.75	0.14	9.98	
	Scenario Average	− 1.72	0.75	0.19	0.15	0.07	− 0.11	0.93	0.42	0.32	− 0.1	0.35	− 0.06	0.31	0.12	0.47	
	Test set Average			− 0.11							0.31				0.12	0.05	
Genetic-based	Avg_GRM_self	3.11	1.59	2.05	0.94	1.41	1.82	0.72	− 0.61	− 0.05	− 0.49	0.46	− 0.37	− 0.06	0.8	1.38	OIL
	Avg_GRM_MinMax	2.08	1.04	1.36	1.04	1.75	1.45	1.77	− 0.7	0.26	− 0.42	− 0.02	0.05	0.16	0.75	0.91	
	PCA_CDmean	0.06	1.49	0.91	0.45	0.84	0.75	1.51	0.71	0.48	0.26	0.41	− 0.49	0.48	0.6	0.35	
Mixed	PLS_CDmean	0.34	0.27	0.89	0.49	0.35	0.47	3.01	1.08	− 0.72	− 0.63	− 0.09	− 0.34	0.38	0.42	1.06	
	Tails_GEGVs	− 11.97	− 3.34	− 1.31	− 1.49	− 2.04	− 4.03	1.52	− 3.67	− 4.23	− 3.9	− 1.5	− 0.22	− 2.00	− 2.92	11.97	
Phenotypic-based	Tails	− 8.1	0.49	− 0.67	− 1.39	0.47	− 1.84	2.78	− 0.08	0.05	1.5	0.62	0.46	0.89	− 0.35	7.77	
	Scenario Average	− 2.41	0.26	0.54	0.01	0.46	− 0.23	1.89	− 0.54	− 0.7	− 0.61	− 0.02	− 0.15	− 0.02	− 0.12	1.1	
	Test set Average			− 0.23							− 0.02				− 0.12	0.01	

The optimization methods are classified based on the type of input they require, i.e. Genetic-based, Phenotypic-based, and Mixed. The “Scenario Average” rows display the average performance of each scenario (test set and candidate set years combination) across optimization methods. The “Test set Average” column provides the average performance within a given test set across candidate sets, while the “Test set Average” rows display the average performance across optimization methods. Additionally, the figure presents the global average performance across scenarios for each optimization method, along with its corresponding variance

### Simultaneous optimization of training set size and composition

Table 3 presents all combinations of methods used for optimizing the TRS size and its composition. The performance of optimization strategies is expressed as a percentage of the predictive ability obtained when the entire candidate set is used to calibrate the models, with values exceeding 100% indicating better optimization performance than using all the data. For all traits, genetic-based methods led to a reduction of the TRS size by 20% with a slight decrease in predictive ability of about 1–2% with the exception of Avg_GRM and Min_GRM for composition optimization in YLD, which led to a loss in predictive ability of around 3–4%. Random sampling resulted in a loss of around 1.5–2%. For YLD, Tails_GEGVs with the size manually set to 60% or optimized with Tails_GEGVs_sd1 led to a dimensionality reduction of 40%, resulting in predictive ability that was greater than using all data in some scenarios and slightly worse in others, averaging to be comparable. Size optimization with Min_GRM followed by composition optimization with Tails resulted in the best performance for GM and OIL, with a 20% reduction in dimensionality and a decrease in performance close to 0.5%.

**Table 3:** Performance of the different combinations of methods for optimizing training set (TRS) size and composition

Trait	TRS Optimization Method			Average TRS size and gain in predictive ability both expressed as a percentage relative to the entire candidate set								
	type	Size	Composition	Test Set year 6			Test Set year 7			Globally		
				Mean	sd	TRS size	Mean	sd	TRS size	Mean	sd	TRS size
YLD	Genetic	Min_GRM	Avg_GRM_self	98.22	2.37	79.29	98.39	2.24	79.81	98.31	2.30	79.55
		Min_GRM	Avg_GRM_MinMax	98.03	2.45	79.29	98.26	2.84	79.81	98.14	2.67	79.55
		Min_GRM	Avg_GRM	96.82	2.44	79.29	97.01	3.28	79.81	96.92	2.92	79.55
		Min_GRM	Min_GRM	97.64	3.29	79.29	95.44	3.22	79.81	96.54	3.46	79.55
		Min_GRM	PCA_CDmean	98.67	2.80	79.29	97.94	2.26	79.81	98.31	2.56	79.55
	Mixed	Min_GRM	PLS_CDmean	99.13	2.24	79.29	98.56	2.54	79.81	98.85	2.43	79.55
		Tails_GEGVs_sd1	Tails_GEGVs	101.41	2.24	60.62	98.95	1.22	60.40	100.18	2.17	60.51
		Manually set 60%	Tails_GEGVs	102.58	2.48	60.00	98.21	1.51	60.00	100.39	3.00	60.00
	Phenotypic	Min_GRM	Tails	98.94	2.44	79.29	98.85	1.88	79.81	98.90	2.14	79.55
		Min_GRM	Random	98.62	2.44	79.29	97.90	2.46	79.81	98.26	2.49	79.55
GM	Genetic	Min_GRM	Avg_GRM_self	98.80	1.56	79.29	98.86	2.12	79.81	98.83	1.89	79.55
		Min_GRM	Avg_GRM_MinMax	98.76	1.48	79.29	98.25	2.48	79.81	98.51	2.10	79.55
		Min_GRM	Avg_GRM	99.27	4.28	79.29	96.98	2.70	79.81	98.13	3.66	79.55
		Min_GRM	Min_GRM	98.43	1.65	79.29	97.70	1.57	79.81	98.06	1.65	79.55
		Min_GRM	PCA_CDmean	98.44	1.82	79.29	98.37	2.65	79.81	98.40	2.31	79.55
	Mixed	Min_GRM	PLS_CDmean	98.25	1.50	79.29	98.21	2.37	79.81	98.23	2.02	79.55
		Tails_GEGVs_sd1	Tails_GEGVs	95.11	2.80	56.19	93.85	3.50	59.36	94.48	3.23	57.77
		Manually set 60%	Tails_GEGVs	96.56	1.23	60.00	95.21	3.14	60.00	95.89	2.53	60.00
	Phenotypic	Min_GRM	Tails	98.68	1.07	79.29	100.37	1.57	79.81	99.52	1.61	79.55
		Min_GRM	Random	98.56	1.59	79.29	98.70	2.84	79.81	98.63	2.36	79.55
OIL	Genetic	Min_GRM	Avg_GRM_self	98.62	1.53	79.29	98.84	2.28	79.81	98.73	1.98	79.55
		Min_GRM	Avg_GRM_MinMax	98.71	1.57	79.29	98.91	2.36	79.81	98.81	2.04	79.55
		Min_GRM	Avg_GRM	95.69	5.11	79.29	100.54	2.94	79.81	98.12	4.83	79.55
		Min_GRM	Min_GRM	96.40	1.80	79.29	100.21	1.65	79.81	98.30	2.58	79.55
		Min_GRM	PCA_CDmean	98.99	1.44	79.29	99.12	2.08	79.81	99.05	1.82	79.55
	Mixed	Min_GRM	PLS_CDmean	98.51	1.58	79.29	99.00	2.15	79.81	98.76	1.93	79.55
		Tails_GEGVs_sd1	Tails_GEGVs	94.26	2.86	62.03	96.38	2.49	62.07	95.32	2.87	62.05
		Manually set 60%	Tails_GEGVs	94.34	2.37	60.00	96.42	2.21	60.00	95.38	2.51	60.00
	Phenotypic	Min_GRM	Tails	98.90	1.11	79.29	99.62	1.62	79.81	99.26	1.44	79.55
		Min_GRM	Random	98.18	1.66	79.29	98.74	2.34	79.81	98.46	2.07	79.55

The optimized training sets for all traits were evaluated using 30 repetitions of gradient boosting machine model. For each test set, the average performance across the different candidate sets tested is displayed. Furthermore, the average for both test sets is in the “Globally” column. The performance values are expressed as a percentage of the predictive ability obtained using the entire candidate set to calibrate the models and the training set size is expressed as a percentage of the candidate set size

**Fig. 5:**
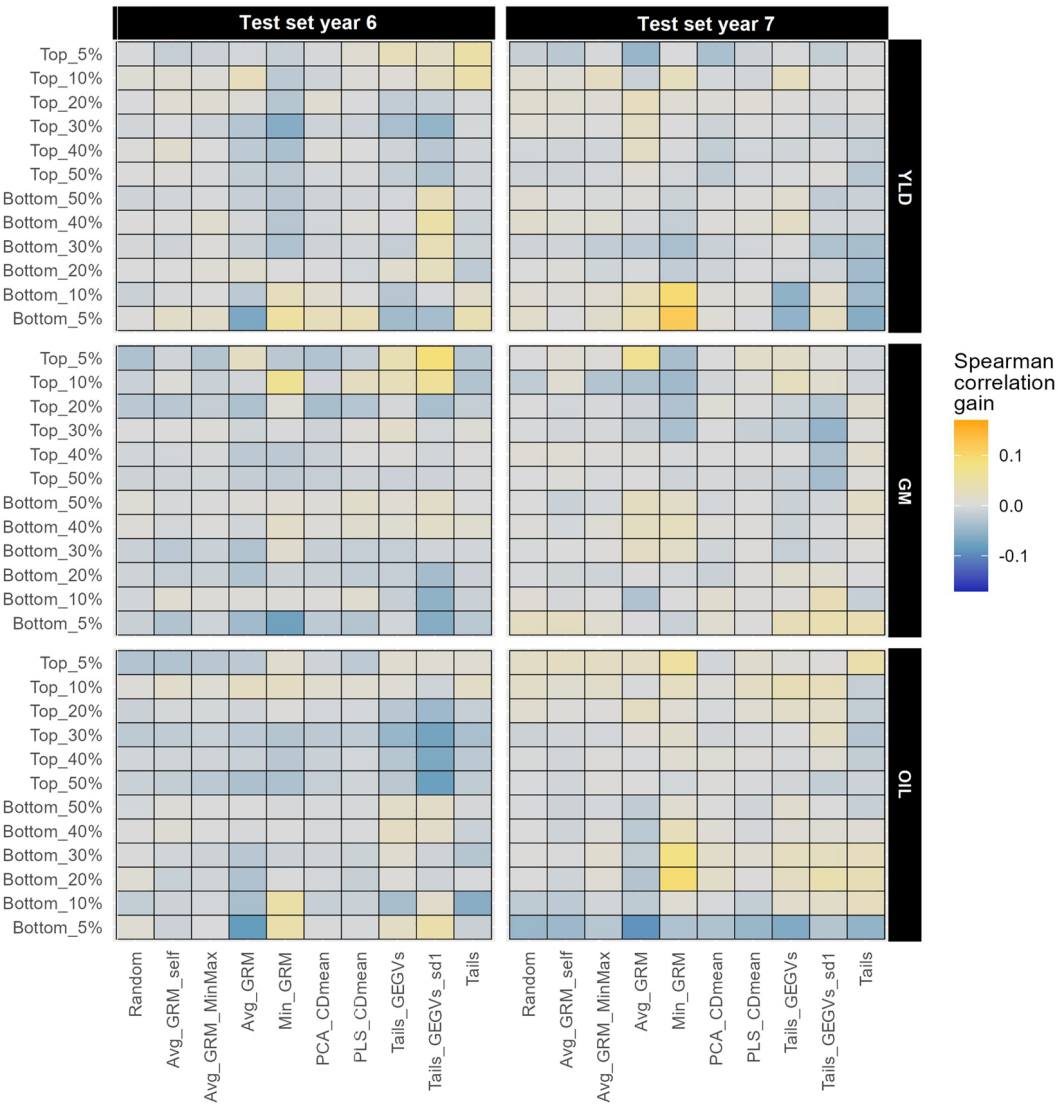
Heatmap showing the average increase (orange) or decrease (blue) of Spearman correlation between test set genotypic values and GEGVs generated by GBM model for multiple training set optimization methods relative to using the entire candidate set to train the model. The average Spearman correlation change is calculated for each trait (displayed on the right-hand side of the vertical axis), optimization method (displayed on the bottom of the horizontal axis), and test set (displayed on the top of the horizontal axis) across repetitions and years included in the candidate set. The Spearman correlation was calculated in several subsets of the test set, created by selecting the highest/lowest genotypic values for the trait of interest (left axis). It is noteworthy that the training set size used for all methods was optimized previously by Min_GRM, except for Tails_GEGVs_sd1, which concurrently optimized the training set size and composition

To further investigate the performance variation of different optimization methods, we analyzed the Spearman correlation between predicted GEGVs (using models calibrated with the TRS) and BLUPs from the first step model (obtained using phenotypic records of the TS) for different segments of the TS representing different proportions of hybrids with high or low genotypic values for each trait (Fig. 5). Among the methods tested, Random sampling, Avg_GRM_self, Avg_GRM_MinMax, PCA_CDmean, and PLS_CDmean, showed consistent  performance across segments, with Spearman correlation values very similar to the ones obtained by models trained using the entire candidate set. In contrast, the other methods were heterogeneous, with better performance for some segments and worse for others. Notably, Tails_GEGVs and Tails_GEGVs_sd1 often demonstrated  better performance than the entire candidate set for the top 5% and 10% segments in all traits and for both TS years. However, no clear pattern was found for Tails, Min_GRM, and Avg_GRM.

### Effect of common parents in training and test sets

**Fig. 6:**
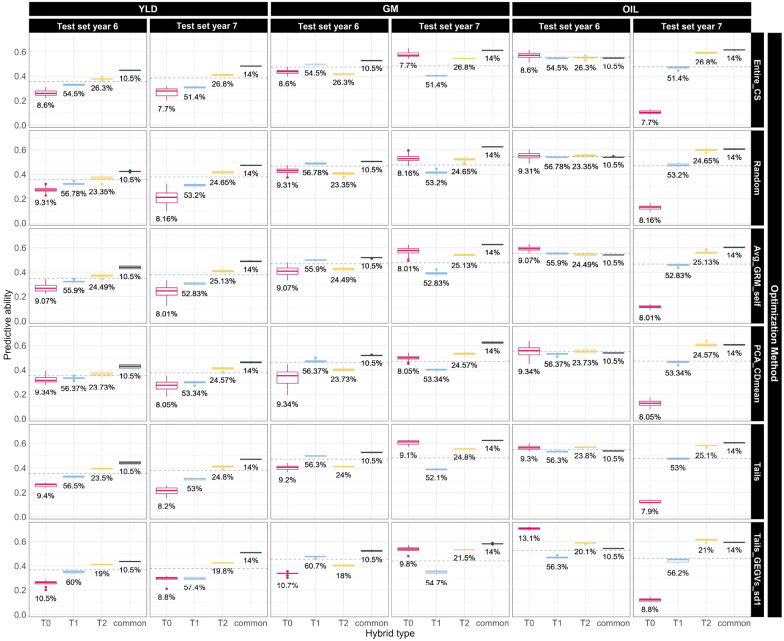
Boxplot of predictive abilities for hybrids across different training set optimization and modelling iterations. The grid displays combinations of traits and test set years (top) and optimization methods (right). Only the best performing optimization methods are shown, and the training set size used was the optimal one found by Min_GRM except for Tail_GEGVs_sd1, which concurrently optimizes size and composition, and Entire_CS, using all available data without optimization. The candidate set considered comprised data from all years preceding the test set. Test set hybrids are categorized as T0, T1, T2 or Common, based on number of common parents in training and test sets. The dashed horizontal line represents the average predictive ability for all hybrids in each scenario. The percentage below each box denotes the proportion of the total test set comprised by the corresponding hybrid type

We classified hybrids in the TS into four types based on how many of their parents were also used as parental lines of TRS hybrids: (i) T0 if neither parent was used, (ii) T1 if one parent was used, (iii) T2 if both parents were used, and (iv) common if the same hybrid combination appeared in both sets. The performance of hybrid types varied depending on the trait and TS used, with the number of years in the TRS having only a minor effect (Additional file 3: Fig. S12). We thus focused on TRS containing all data older than the TS in Fig. 6, while varying the trait, TS year, and TRS optimization method. For YLD, T0 hybrids had the lowest predictive ability in almost all cases, followed by T1, T2, and common hybrids. This trend was also observed in OIL for TS year 7, but when the TS was year 6, all hybrid types tended to perform similarly. In GM, common hybrids achieved the highest predictive ability. T0 and T2 were usually similar and inferior to common hybrids, while T1 was better than them for TS year 6 and worse for TS year 7.

Figure 6 also illustrates the impact of optimization methods on hybrid classification. T1, T2, and common hybrids consistently exhibited low dispersion across all optimization methods, while T0 hybrids showed relatively large dispersion for random sampling and TrainSel methods (Avg_GRM_self and PCA_CDmean). However, T0 hybrids exhibited smaller dispersion for Tails_GEGVs_sd1, Tails and Entire_CS. In the latter three cases, all dispersion is caused by the random start of GBM model across iterations. However, in random sampling and TrainSel methods, the random starting point in the optimization process influences the final composition of the TRS, thereby increasing dispersion. It is worth noting that, while Tails, Tails_GEGVs_sd1 and Entire_CS present low dispersion, Tails_GEGVs_sd1 has the lowest one, as clearly observed in YLD, TS year 7. The impact of TRS optimization on the relative predictive ability of the different hybrid types was generally negligible. However, Tails_GEGVs_sd1 increased the performance of T0 hybrids in certain scenarios (YLD, TS year 7; OIL TS year 6) and reduced it in GM, TS year 6.

## Discussion

In this study, we investigate the impact of incorporating older historical data into the TRS for improving GS accuracy in sunflower breeding. While previous studies have focused on optimizing TRS within a year and a generation, the optimization of historical data in an across-year and across-generation scenario for efficient utilization in hybrid crops has not been extensively explored. We aim to fill this gap by evaluating the performance of different methods for optimizing TRS size and composition using genotypic and phenotypic historical data from a sunflower breeding program. This study is unique as it provides a rare opportunity to investigate the impact of historical data on genomic prediction using large-scale empirical data from a commercial sunflower breeding program.

In this work, we have focused on prediction accuracy to evaluate the performance of optimization. While maximizing accuracy is a key goal in genomic selection, particularly for early-stage selection, it is crucial to maintain a balanced approach. In plant breeding, unlike animal breeding, the final selection of new varieties often involves extensive multi-environment trials where genomic selection has less impact than other tools employed. Furthermore, other major drivers of genetic gain in genomic selection such as intensity of selection and generation interval were out of the scope of this study. However, an increased GS accuracy through optimized historical data usage could allow to implement GS in earlier breeding stages, which can lead to improvements on intensity of selection and generation interval.

### Optimization of the years to be included in the training set

The inclusion of older historical data in the TRS can have varying effects, including increased TRS size, which usually results in enhanced diversity and predictive ability [19, 22–28, 49]. However, incorporating older data that is narrowly related to the TS and may have different linkage disequilibrium patterns can be detrimental to predictive ability [50]. Additionally, as noted by Bernal-Vasquez et al. [44] and Schrag et al. [46], environmental effects may differ between older and more recent data. Including years with low heritability increases the noise in the data and reduces predictive ability. Consequently, determining the optimal number of older years to include in the TRS involves a trade-off that we leveraged through multi-objective optimization (Fig. 2). It is important to note that this kind of optimization is extremely fast and computational time will not be a limiting factor regardless of the dimensionality of the data. More details are available in Additional file 3, Note 6.

This type of optimization approach yields a set of non-dominated solutions forming a three-dimensional Pareto front [18–20]. Selecting the best option from this set of non-dominated solutions is a critical step and requires additional criteria (for more information, refer to Additional file 3, Note 7). Our findings indicate that maximizing diversity was the most important factor, which is consistent with existing literature [23, 25, 48]. Moreover, prioritizing high diversity implicitly favors larger TRS that encompass a greater number of years. This is crucial for accurate estimation of year effects and the removal of environmental effects during the initial modeling step [44, 46]. Interestingly, our results (see additional file 3: Figs. S4–S8 A, B, D) highlight the importance of selecting solutions with a higher number of years over solutions with slightly higher relationship to the TS and heritability. Notably, it is worth mentioning that the optimization process never selected a combination of years that included year 1, as its inclusion led to a reduction in average diversity (Fig. 2A), likely caused by a redundancy between the hybrids found in year 1 and subsequent years.

Regarding heritability, low heritability posed challenges in accurately estimating genetic effects in the first step model, leading to increased noise in the data and potentially reducing predictive ability. In the second step model, low heritability is frequently associated with a higher proportion of variance explained by non-additive genetic effects, which are more difficult to estimate than additive ones, further compromising predictive ability [75, 76]. This is exemplified by year 3 in the YLD trait, where the very low heritability often made excluding it from the TRS the optimal strategy (Fig. 3, Table 1). In contrast, the relationship to the TS emerged as the least important variable, which contradicts findings in the literature [50]. This discrepancy is likely due  to the dataset used in our study, where the TRS and TS mostly overlapped in the genetic space (Additional file 3: Fig. S13). Consequently, all TRS years were sufficiently related to the TS and provided informative data for the GS model.

### Training set optimization for fixed years in training and test sets

**Fig. 7:**
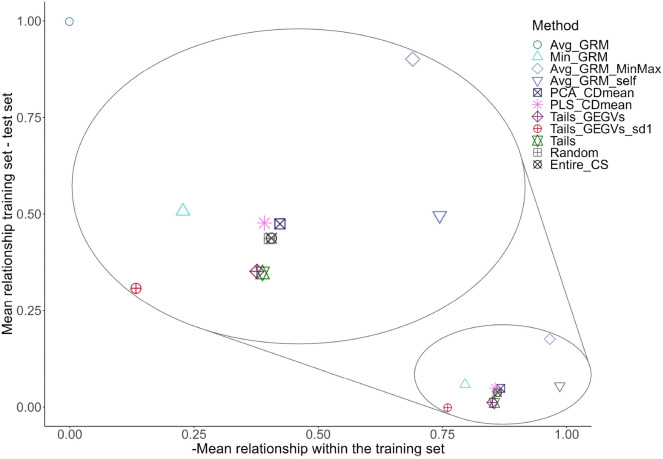
Trade-off between the average additive relationship between the training and test sets (vertical axis) and the opposite value of  the average additive relationship within the training set (indicating training set diversity in horizontal axis). The values in both axes have been normalized between 0 and 1. Each point corresponds to a different method for optimizing training set composition using the optimal training set size found by Min_GRM (with the exception of Tails_GEGVs_sd1, which simultaneously optimizes training set size and composition). The values obtained for each method correspond to the average across all scenarios. For visualization purposes, the content within the large ellipse is a zoom-in of the small ellipse. The position of the optimization methods within the small ellipse is their true location

In the present study, we observed a trend in predictive ability that is commonly reported in the literature, where enlarging the TRS initially leads to a rapid increase in performance, but tends to plateau for larger TRS sizes [19, 22–28]. However, in our results (Fig. 4), this trend was not particularly pronounced. One explanation for this could be the high dimensionality of the data used in our study, where even the smallest TRS considered (20% of the candidate set) contained around 400 to 2000 hybrids, which is a large TRS in absolute terms when compared with the TRS commonly used in the literature [26, 27, 48, 51–53]. The small differences in performance observed between different optimization methods (Table 4) can also be attributed to this, as differences in predictive ability are typically more pronounced for smaller TRS sizes [19, 22–27].

Contrary to our expectations [19, 22], targeted methods did not outperform untargeted ones (Fig. 4, Table 2). In our dataset, the candidate set and TS were highly related genetically, occupying a similar portion of the genetic space (Additional file 3: Fig. S13). This makes targeted optimization less critical, as any diverse sampling of the candidate set will be strongly related to the TS. CDmean, which has been shown to be the best-performing targeted method [19], underperformed in our work due to the need for dimensionality reduction to accelerate computations (more details about computational time of all methods are available in Additional file 3, Note 6). PCA_CDmean and PLS_CDmean sampled TRS with less diversity and lower relationship to the TS than Avg_GRM_self, despite being targeted methods (Fig. 7).

Our results showed that Tails_GEGVs_sd1 was the best-performing method for predicting YLD, resulting in a 40% dimensionality reduction and a slight performance gain. This is consistent with previous findings [54] that a TRS composed of genotypes with both the best and worst breeding values performs better than only considering the genotypes with the highest breeding values. Tails_GEGVs excludes hybrids with intermediate performance values, leaving genotypes that tend to contain alleles with effects of the same sign, making it easier to estimate their effects in the presence of non-additive interactions. This would explain why this method performed better for YLD, which has the lowest additive-to-dominance variance ratio (Table 4). The extreme hybrids sampled by Tails_GEGVs need to have their dominance effects in the same direction for most loci, which probably helps to differentiate additive and dominance effects. Supporting this, prior research has demonstrated that non-additive variance can be better captured in the case of extreme allelic frequencies [55]. The need to remove the hybrids with intermediate genotypic values may explain why the best performance was usually reached at smaller TRS sizes compared to other methods (Fig. 4A). Moreover, Tails_GEGVs does not maximize the genetic diversity of the TRS (as clearly seen in Fig. 7), but it maximizes the diversity of alleles with an important effect on the trait of interest by sampling hybrids with extreme values, indirectly considering marker effects. Finally, as Tails_GEGVs uses phenotypic information, it is influenced by environmental effects, which have a large effect on low heritability traits like YLD, especially in across-year predictions. This may explain the inconsistent performance of this method across scenarios and traits.

Regarding optimization of TRS size [19, 28], we developed Min_GRM size optimization, which was able to consistently find the optimal value in all scenarios, resulting in a 20% dimensionality reduction with an average accuracy loss of around 1.50% (Table 3). Min_GRM is able to identify the genotypes in the candidate set with a high genetic relationship to the TS. This has the disadvantage of not considering the diversity within the TRS, which can be extremely detrimental to GS performance, as happened to Avg_GRM in [19]. Min_GRM was designed to follow a similar concept as Avg_GRM while being able to better preserve diversity within the TRS [53], which coincides with the results observed in Fig. 7. Furthermore, Avg_GRM and Min_GRM outperformed CDmean in Lemeunier et al. [53]. The likely reason behind it is the fact that, in Lemeunier et al. [53], the dataset used was characterized by having a TS that occupied only a subset of the genetic space spanned by the candidate set. Therefore, if a suitable TRS size is set, it is possible for Avg_GRM and Min_GRM to find all genotypes in the candidate set that overlap with the genetic space of the TS, i.e. all relevant diversity is selected. This would be the optimal size, which can be efficiently found using the Min_GRM optimization developed here (Table 6, Additional file 3: Fig. S9). In our work, the candidate set and TS occupied mostly the same part of the genetic space (Additional file 3: Fig. S13), and as a result, the optimal size was very large. Further work is required to test Min_GRM size optimization in datasets with a distribution of TRS and TS in the genetic space similar to the one in Lemeunier et al. [53]. In that scenario, we hypothesize that a plot similar to Additional file 3: Fig. S9 would have the shape of the sum of as many different sigmoidal curves as clusters are in the population, and the optimal size would be the second inflection point of the first one.

Finally, we optimized the TRS size and composition and examined the predictive ability of various methods in different segments of the TS. We found that, while many methods had similar predictive abilities on the entire TS (Table 3), some methods performed better in certain segments (Fig. 5). For instance, we observed that methods that maximized diversity within the TRS (Avg_GRM_self, Avg_GRM_MinMax, PCA_CDmean and PLS_CDmean), as well as random sampling, resulted in homogeneous performance across all segments of the TS. This highlights the importance of diversity for consistent predictions. However, these consistent methods rarely outperformed those using all data. In contrast, methods that did not maximize diversity (Avg_GRM, Min_GRM, Tails_GEGVs, Tails_GEGVs_sd1, and Tails), performed substantially better than using all data in some segments, while performing worse for others. This could be leveraged to improve predictions for key segments of the TS (e.g. hybrids with the highest or lowest genotypic values). However, this is only possible if a method consistently outperforms all data for the same segment of interest in all situations. This was usually true for Tails_GEGVs and Tails_GEGVs_sd1 for the top 5 and 10% hybrids in Fig. 5. Further research in different datasets is needed to explore this phenomenon.

### Effect of common parents in training and test sets

In the literature, it has been described that the accuracy of predictions for a hybrid is heavily dependent on how many of its parents have also acted as parents in the TRS [56, 57]. We explored this and its interaction with TRS optimization in Figs. 6, Additional file 3: Fig. S12. The performance of different kinds of hybrids was highly influenced by the trait and TS year, as shown in Fig. 6. Generally an increasing number of common parents between the TRS and TS resulted in higher predictive ability, which is consistent with previous literature [56]. However, in the case of GM and OIL for TS year 6, the opposite was true. As discussed in [57], the prediction of T0 hybrids greatly benefits from the inclusion of SCA in the model, emphasizing the importance of non-additive effects in predicting these hybrids. The GBM model used in this study can capture a wide range of non-additive effects, which may explain the high predictive ability for T0 hybrids in certain scenarios. Furthermore, differential genotype by environment interactions in the two TS years may partially account for the different patterns observed. To further explore the impact of the TS year, we created Additional file 3: Fig. S14, which displays the distribution of the different types of hybrids in the genetic space for both TS years. Interestingly, while T1, T2, and common hybrids occupied most of the genetic space in in both TS years, T0 hybrids were mainly clustered in four regions in the bottom and bottom-right of the plot in TS year 7, while they were more prevalent in the top and right of the plot in TS year 6. These differences explain why the TS year had a significant impact on the performance of T0 hybrids across all traits. Furthermore, T0 hybrids were the least numerous group (Fig. 6), ranging between 7.7 and 13.1% of the TS depending on the scenario (around 300 to 400 hybrids). Their relatively small sample size may have also played a role in the high variability of their performance across scenarios.

In terms of the dispersion of predictive ability within each scenario, T1, T2, and common hybrids demonstrated exceptional consistency across all scenarios (Fig. 6). However, the dispersion of T0 hybrids varied significantly among optimization methods. Methods that relied on a random start (such as Random sampling, Avg_GRM_self, and PCA_CDmean) exhibited considerable dispersion, suggesting that slight variations in the TRS caused substantial differences in T0 hybrid predictions, particularly for low heritability traits such as YLD and GM. Although the dispersion was lower for Tails and when all available data was utilized as a TRS (Entire_CS in Fig. 6), it was still higher than for other hybrid types. This highlights that the GBM model struggled to achieve consistent results across random starts, further indicating the difficulty of predicting T0 hybrids. In contrast, when the Tails_GEGVs_sd1 optimization was performed, the dispersion for T0 hybrids was negligible, supporting our hypothesis that this method removes confounding effects in the training data.

## Conclusions

This study focused on optimizing the utilization of historical data for genomic prediction in a large-scale commercial hybrid sunflower dataset. Through the use of multi-objective optimization, we balanced the variables of diversity, heritability, and the relationship between the TRS and TS. This allowed us to consistently identify the optimal combination of years to be included in the TRS, prioritizing high diversity while also considering the number of different years selected and maintaining high average heritability and relationship to the TS. In terms of optimization methods, the Min_GRM approach proved effective in determining the optimal size of the TRS. It could be combined with other methods for optimizing the composition, with Tails emerging as the best-performing method. This resulted in a 20% reduction in dimensionality while only slightly impacting predictive ability. While Tails_GEGVs showed potential for traits with low heritability and high complexity, outperforming the use of all data for YLD and facilitating more consistent modeling for T0 hybrids, their predictive performance varied across different scenarios. This inconsistency underscores the need for further research to fully comprehend the underlying reasons. Additionally, our study revealed that, when the TS is segmented based on genotypic values, a highly diverse TRS results in uniform predictive ability across all segments. In contrast, Tails_GEGVs had the ability to exploit heterogeneity across segments, enhancing performance in key areas. However, the performance improvement was not consistent across all scenarios, indicating room for further optimization. These observations offer crucial insights for the optimal use of historical data in breeding programs, while also pointing out the areas where additional investigation is required. Further validation is necessary for self-pollinated crops and breeding programs with different population structures to fully assess its applicability. Moreover, a simulation study could provide valuable insights into the factors that influence Tails_GEGVs performance and lead to its inconsistency across traits.

## Methods

### Plant material

In this study, we utilized a private dataset that contained phenotypic observations of 32,489 sunflower hybrids grown in more than 10 locations over a period of 7 years, with a slight but not significant imbalance in the number of locations tested per year. Due to confidentiality agreements, we are precluded from identifying the exact number of locations, the years and the specifics of the plant material and dataset. Instead, we denote the years as year 1 (oldest) through year 7 (most recent). We evaluated 3 traits, grain yield (YLD), grain moisture (GM), and percentage of oil (OIL).

We used a DNA marker chip consisting of 17,270 markers to genotype 3171 female and 5151 male parental lines. After excluding heterozygous loci, we predicted the genotype of the hybrid offspring from their parental lines. We used “snpReady” R package version 0.9.6 [58] to perform quality control. We removed loci with a minor allele frequency smaller than 0.01 or with more than 20% missing data, and hybrids with over 50% missing data. The remaining missing values were imputed using the k-nearest neighbors method in the “impute” R package, version 1.70.0 [59]. We obtained 16,492 hybrids with BLUPs for the three traits and complete data for 10,145 markers after quality control.

Table 4 provides additional details about the traits considered, including the broad sense Cullis heritability [73] and additive/dominance genetic variance for each trait. For further information on how we obtained these estimates, please refer to Note 3 in the Additional file 3.

**Table 4:** Broad sense Cullis heritability (${\text{H}}^{2}$), variance of female general combining ability ($\sigma$^2^_GCAf_), variance of male general combining ability ($\sigma$^2^_GCAm_), and variance of specific combining ability ($\sigma$^2^_SCA_) for each trait across all years and locations

Trait		$\text {H}^2$	$\sigma$^2^_GCAf_	$\sigma$^2^_GCAm_	$\sigma$^2^_SCA_	Ratio $\sigma ^{2}_{a}/\sigma ^{2}_{d}$
YLD		0.43	4.28	3.15	2.27	3.27
GM		0.46	0.47	0.28	0.11	6.82
OIL		0.64	1.08	0.90	0.31	6.39

In addition, we calculated the ratio of additive and dominance variances (Ratio $\sigma ^{2}_{a}/\sigma ^{2}_{d}$) as $(\sigma ^{2}_{GCAf} + \sigma ^{2}_{GCAm})/\sigma ^{2}_{SCA}$. The traits evaluated were grain yield (YLD), grain moisture (GM), and percentage of oil (OIL). Further details on these calculations can be found in the Additional file 3, Note 3

### Methods

#### **Optimization pipeline**

**Fig. 8:**
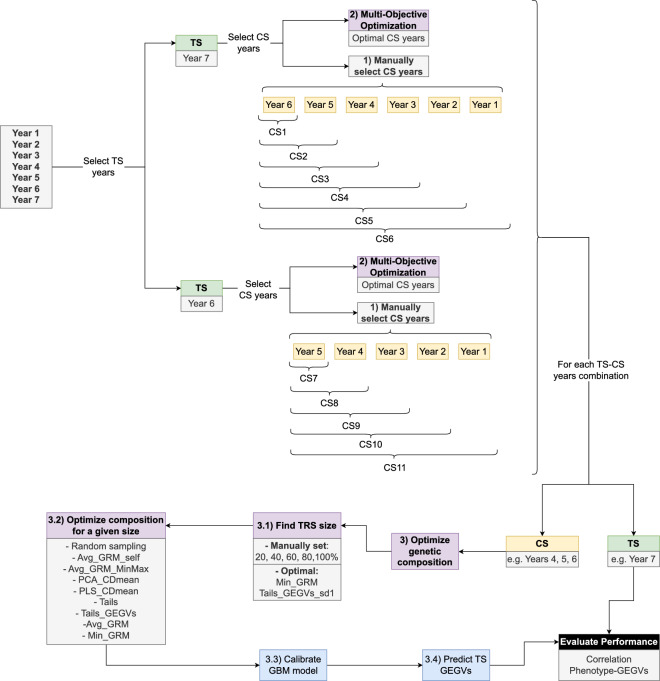
Summary of the methodology used in this work. From the seven years available, two have been selected as test sets (TS) and for each one the candidate sets (CS) can be selected manually of through optimization. For a given combination of TS-CS years, further optimization is possible to find the actual hybrids used in the training set (TRS), which is a subset of the CS. The TRS is subsequently used to train the gradient boosting machine (GBM) model employed to evaluate optimization performance. It is important to note that, when the TRS size is set to 100% of the CS, no optimization can take place, as the entire CS would be used as TRS

The optimization pipeline can be described in three steps, summarized in Fig. 8:

1. Study the year effect in eleven scenarios with different years in the candidate set and TS (Table 5). In this step, all data in the candidate set years is used to train the GBLUP and GBM models with the aim of elucidating whether or not including increasingly older historical data in the TRS improves predictive ability (i.e., in Fig. 8, this corresponds to TRS size = 100% of candidate set).
2. Perform multi-objective optimization to identify the best combination of years to include in the TRS and compare its results with the ones obtained in the previous step. It is important to note that we did not constrain the optimization to only considering the scenarios in Table 5.
3. Optimize the genetic composition of the TRS in the same 11 scenarios as before (Table 5), i.e., from all the hybrids phenotyped in the candidate set years, a subset is taken to act as the actual TRS. Within each scenario, the first step was finding the desired TRS size. To that end, we used size optimization methods (Min_GRM and Tails_GEGVs_sd1) and we also tested sizes manually set (20, 40, 60 and 80% of the entire candidate set). For each size, all methods for optimizing TRS composition were used and they were evaluated using the predictive ability of GS models calibrated with the corresponding optimized TRS. We performed 10 repetitions for optimization methods based on TrainSel, version 2.0 [60] (see Table 6 and Additional file 3, Note 6) and we used 10 repetitions of the GBM model for each TRS to account for the influence of the random start. GBLUP model was not employed in this step due to issues with computational time caused by the high dimensionality of the data (number of genotypes larger than number of markers)

**Table 5:** Combinations of years in the candidate and test sets in which the different training set optimization methods were tested. For each test set year, the candidate set is initially composed of the previous year, and older years are progressively included, e.g. 5-3 indicates that the candidate set contains all data from years 3, 4, and 5. For a given combination of years in the candidate set, optimization can be used to find an optimal subset of all hybrids tested in said years. This subset then becomes the training set. The optimized training sets are subsequently used to calibrate genomic selection models. Finally, the predictive ability of the model in the test set is used to evaluate the performance of the optimization of its training set

Candidate set years						Test set year
−	5	5-4	5-3	5-2	5-1	6
6	6-5	6-4	6-3	6-2	6-1	7

#### **Genomic selection models**

For all models, we used a two-step approach. In the first step, we removed environmental effects and estimated Best Linear Unbiased Predictions (BLUPs) for each hybrid. In the second step, we utilized the BLUPs and genotypic information as inputs to train the model.

*First step model:*

1 $$\begin{aligned} {\varvec{{y}}} = {\textbf {1}}\mu + X\varvec{\beta } + Z{\textbf {g}} + \varvec{\epsilon } \end{aligned}$$

where ***y*** is a vector of hybrid phenotypic records, **1** is a vector of ones, $\mu$ is the intercept, $\varvec{\beta }$ is a vector of fixed effects corresponding to environmental effects (year:location combinations), ***g*** is a vector of best linear unbiased predictors (BLUPs) for the random effects of the genotype, $\varvec{\epsilon }$ is a vector of residual random effects and *X* and *Z* are design matrices for the environmental and genotypic effects respectively. ***g*** and $\varvec{\epsilon }$ follow a multivariate normal distribution of mean **0** and variance-covariance structure $I\sigma ^{2}_{g}$ and $I\sigma ^{2}_{e}$ respectively, where $\sigma ^{2}_{g}$ is the genetic variance, $\sigma ^{2}_{e}$ is the residual variance and *I* is the identity matrix of the appropriate dimensions. The BLUPs in ***g*** (of a much lower dimensionality than the observations in ***y***) will be used for all subsequent analyses. While the primary advantage of BLUPs lies in their ability to model genetic relationships by the integration of variance-covariance structure instead of an identity matrix, our decision to use an identity matrix was guided by the need to balance computational feasibility with the size of our dataset. The genomic data will be employed in the second step models. In acknowledging the potential of alternative approaches, it is important to note that employing BLUEs in the initial stage would be a viable method. This approach is currently under investigation in our ongoing research, where we are working towards implementing a fully efficient model [61, 62]. Furthermore, it could be discussed whether the environmental effect should be fixed or random. The environment in this dataset can be regarded as a stochastic process, which would be better modelled as a random effect. However, the number of levels for the environment can be relatively small in some of the scenarios, potentially compromising the estimation of the variance component. Setting the environmental effect as fixed removes the number of environmental levels as a source of variation for model performance across scenarios. Finally, we have assumed homogeneous residuals, while heterogeneous residuals across locations would have been more realistic. The reason for it is that this allowed us to fit the model in with the extremely computationally efficient lme4 R package, version 1.1-34 [63]. This was critical due to the large dimensionality of our dataset.

It is important to mention that the first step was carried out separately for the TS and the candidate set (set of hybrids from which the TRS will be sampled by optimization methods) to ensure that no information from the TS was included in the TRS. When using data from only one year for the first step, the environmental fixed effects refer to the location rather than the year and location combinations.

Two different models were used as a second step. For a detailed comparison of their performance, please refer to Additional file 3, Note 4.

*GBLUP*: 

A linear mixed model based on the general combining ability (GCA) and specific combining ability (SCA) of the parental lines was used. As a result, it can take into account additive and dominance effects:

2 $$\begin{aligned} {\varvec{{y}}} = {\textbf {1}}\mu + Z_{1}{} {\textbf {f}} + Z_{2}{} {\textbf {m}} + Z_{3}{} {\textbf {h}} + \varvec{\epsilon } \end{aligned}$$

Where ***y*** is a vector containing the BLUPs obtained in the first step model, **1** is a vector of ones, $\mu$ is the intercept, ${\textbf {f}} \sim N({\textbf {0}}, \sigma ^{2}_{f}G_{f})$ is the vector of random effects for the GCA of the female parents, ${\textbf {m}} \sim N({\textbf {0}}, \sigma ^{2}_{m}G_{m})$ is the vector of random effects for the GCA of the male parents, ${\textbf {h}} \sim N({\textbf {0}}, \sigma ^{2}_{h}H)$ is the vector of random effects for the SCA for the hybrids and $\varvec{\epsilon } \sim N({\textbf {0}}, \sigma ^{2}_{e}I)$ is the vector of residuals. $G_{f}$ and $G_{m}$ are the additive relationship matrices for males and females respectively calculated from genomic data using the VanRaden method [64] and *H* is the dominance relationship matrix calculated from the marker data of the hybrids [65]. $\sigma ^{2}_{f}$, $\sigma ^{2}_{m}$, $\sigma ^{2}_{h}$ and $\sigma ^{2}_{e}$ are the variances for females, males, hybrids and residuals respectively. This model was implemented using the Sommer R package, version 4.1.7 [66]. For further details about the calculation of the relationship matrices, see Additional file 3, Note 1.

*Gradient boosting machine* (GBM):

This model uses an ensemble of weak learners (decision trees) sequentially built in such a way that each tree is fitted on the residuals of the previous ones and minimizes them [67]. The input of this model is a vector of BLUPs for the hybrids in the TRS and its corresponding marker matrix. An important previous step to maximize the performance of this model and avoid overfitting is the tuning of its hyperparameters, which was carried out performing grid search and cross validation within the candidate set (For more details see Additional file 3, Note 4). This model is nonlinear and, as a result, it can implicitly consider non-additive effects such as dominance and epistasis. We implemented it using the R xgboost package, version 1.7.3.1 [68].

#### **Optimization of the years to be included into the training set**

The first step when working with historical data is determining from which years the TRS data should originate. To that end, we developed a multi-objective optimization using TrainSel, version 2.0 [69] heuristic and simultaneously maximizing TRS diversity, relationship to the TS and heritability:

3 $$\begin{gathered} {\text{Diverstiy}} = - mean\,\left( {G_{{TRS;TRS}} } \right) \hfill \\ {\text{Relationship to TS}}\,{\text{ = }}\,mean\,\left( {G_{{TRS;TS}} } \right) \hfill \\ {\text{Heritability}} = mean\left( {{\varvec{H}}_{{TRS}}^{{\mathbf{2}}} } \right) \hfill \\ \end{gathered}$$

Where mean($\cdot$) indicates the average of a vector or matrix, *G* is the additive relationship matrix between hybrids, ${\varvec{H}}^{{\mathbf{2}}}$ is a vector containing the heritability within each TRS year and a subindex indicates that a subset of the vector or matrix is taken, with *TRS* and *TS* referring to the years in the training and test sets respectively.

#### **Training set optimization methods**

For a given combination of years in the TRS, its genetic composition can be further optimized by several TRS optimization methods. In this scenario, all data from the combination of years of interest conforms a candidate set and TRS optimization methods are used to find an optimal subset of it to be used as the actual TRS.

In this study, we based the classification of the optimization algorithms on the input data. We labeled them as “genetic-based” methods if they utilized only marker data, “phenotypic-based” if they utilized only BLUPs from the first step model, and “mixed” methods if they utilized both. We also labeled them as “targeted” if they required marker data from the TS and “untargeted” if they did not [18, 22]. We implemented trait-specific optimization strategies for phenotypic-based and mixed methods. While we could use all methods to optimize the composition of the TRS, only certain methods were appropriate for optimizing its size. We provide the equations for these methods in detail in Table 6.

##### **Genetic-based methods**

*PCA_CDmean (targeted).* CDmean [21] can be considered the gold standard for TRS optimization, but its high computational cost [19] makes its implementation in industrial-scale datasets difficult. Here, we used an implementation accelerated by principal component analysis (PCA) on the marker data. This implementation is equivalent to CDMEAN2 in [70].

*Avg_GRM_self (untargeted)*. This method minimizes the average relationship within the TRS to maximize variability [19].

*Avg_GRM_MinMax (targeted).* It minimizes average relationship within the TRS similarly to Avg_GRM_self but it also maximizes the average relationship between TRS and TS [19].

*Avg_GRM (targeted)*. Maximize the average relationship between TRS and TS (see Table 6 or OPT_MEAN in [53] for more details).

*Min_GRM (targeted)*. Maximize the minimum relationship between the individuals of the TRS and any individual in the TS. (see Table 6 or OPT_MIN in [53] for more details). Min_GRM has solely been utilized in literature to optimize the TRS composition. However, we applied it to optimize the size of the TRS as well. Testing all possible TRS sizes yielded a sigmoidal curve (Additional file 3: Fig. S9), where the second inflexion point corresponds to the optimal TRS size. Once the optimal size is determined, the TRS composition can be optimized using Min_GRM or any other method. More information can be found at Table 6 and Additional file 3: Fig. S9.

##### **Phenotypic-based methods**

*Tails (untargeted)*. To obtain a TRS of a predetermined size $n_{TRS}$, we employed a selection procedure based on the BLUPs from the first step model. Specifically, we chose the $n_{TRS}/2$ hybrids with the lowest BLUPs and the $n_{TRS}/2$ hybrids with the highest BLUPs from the candidate set [47, 71]

##### **Mixed methods**

*PLS_CDmean (targeted)*. Similar to PCA_CDmean but instead of relying on principal component analysis to reduce dimensionality, it uses partial least squares (PLS). More details can be found in Additional file 3, Note 5.

*Tails_GEGVs (untargeted)*. We used GBLUP to compute the GEGVs of all hybrids in the candidate set, followed by Tails optimization using the GEGVs instead of BLUPs from the first step model, in accordance with previous studies [47, 71]. We investigated multiple methods to optimize TRS size and composition simultaneously using Tails_GEGVs, using concepts such as nucleotide diversity [72] (Additional file 3, Note 2, Table S1), and found in preliminary analyses that Tails_GEGVs_sd1 performed the best. This strategy involves selecting individuals with GEGVs below $(mean - \alpha \cdot sd)$ for the lower tail and above $(mean + \alpha \cdot sd)$ for the upper tail, in a scaled GEGVs distribution with $sd=1$ and $\mu =0$. The value of $\alpha$ was set to 0.5 based on previous analyses.

**Table 6:** This table summarizes the TRS optimization methods employed in this study, indicating their purpose (either optimization of size or composition) and type (whether genetic-based, phenotypic-based, or mixed and targeted or untargeted)

Method	Purpose	Type	Mechanism
$$\begin{array}{cc} \text {PCA\_CDmean/} \\ \text {PLS\_CDmean} \end{array}$$	Composition	$$\begin{array}{cc} \text {Genetic/Mixed} \\ \text {targeted} \end{array}$$	$$\begin{array}{cc} D_1 = diag(X_{TS;All}X'_{TS;All}) \\ X_1 = X'_{TRS;All}X_{TRS;All};\\ X_2 = (X_1+I\lambda )^{-1} \\ D_2 = diag(X_{TS;All}X_2X_1X_2X'_{TS;All}) \\ \text {\textit{CDmean}} = -sum(D_2/D_1)/n_{TS} \\ argmax(CDmean) \end{array}$$
Avg_GRM_self	Composition	$$\begin{array}{cc} \text {Genetic} \\ \text {untargeted} \end{array}$$	$$\begin{array}{cc} \text {\textit{Avg\_GRM\_self}} = -mean(G_{TRS;TRS})\\ argmax(\text {\textit{Avg\_GRM\_self}}) \end{array}$$
Avg_GRM_MinMax	Composition	$$\begin{array}{cc} \text {Genetic} \\ \text {targeted} \end{array}$$	$$\begin{array}{cc} \text {\textit{Avg\_GRM\_MinMax}} = mean(G_{TRS;TS}) -mean(G_{TRS;TRS})\\ argmax(\text {\textit{Avg\_GRM\_MinMax}}) \end{array}$$
Avg_GRM	Composition	$$\begin{array}{cc} \text {Genetic} \\ \text {targeted} \end{array}$$	$$\begin{array}{cc} \text {\textit{Avg\_GRM}}_i = mean(G_{i;TS}) \end{array}$$ $$\begin{array}{l} \text {1) Compute \textit{Avg\_GRM} for all hybrids in the candidate set} \\ \text {2) Select for the TRS the } n_{TRS} \text { hybrids with the highest} \\ \text {\textit{Avg\_GRM} values} \end{array}$$
$$\begin{array}{c} \text {Min\_GRM} \end{array}$$	Composition	$$\begin{array}{cc} \text {Genetic} \\ \text {targeted} \end{array}$$	$$\begin{array}{cc} \text {\textit{Min\_GRM}}_i = min(G_{i;TS}) \end{array}$$ $$\begin{array}{l} \text {1) Compute \textit{Min\_GRM} for all hybrids in the candidate set} \\ \text {2) Select for the TRS the } n_{TRS} \text { hybrids with the highest} \\ \text {\textit{Min\_GRM} values} \end{array}$$
	Size	$$\begin{array}{cc} \text {Genetic} \\ \text {targeted} \end{array}$$	$$\begin{array}{l} \text {1) Optimize composition of training sets of increasing size using} \\ \text {\textit{Min\_GRM}. Sizes tested range from 1 to entire candidate set} \\ \text {2) The \textit{fitness} value for every TRS is the smallest \textit{Min\_GRM}} \\ \text {value among its hybrids} \\ \text {3) Plot \textit{TRS size} against \textit{-fitness} as in Figure S9} \\ \text {4) Fit sigmoidal function to the plot} \\ \text {5) Optimal TRS size is the one corresponding to the second} \\ \text {inflexion point of the sigmoidal} \end{array}$$
Tails	Composition	$$\begin{array}{cc} \text {Phenotypic} \\ \text {untargeted} \end{array}$$	$$\begin{array}{l} \text {1) Rank hybrids according to their genotypic values} \\ \text {2) Select } \frac{n_{TRS}}{2} \text { hybrids with highest genotypic values} \\ \text {and } \frac{n_{TRS}}{2} \text { hybrids with lowest genotypic values} \end{array}$$
Tails_GEGVs	Composition	$$\begin{array}{cc} \text {Mixed} \\ \text {untargeted} \end{array}$$	$$\begin{array}{l} \text {1) Rank hybrids according to their GEGVs} \\ \text {2) Select } \frac{n_{TRS}}{2} \text { hybrids with highest GEGVs} \\ \text {and } \frac{n_{TRS}}{2} \text { hybrids with lowest GEGVs} \end{array}$$
Tails_GEGVs_sd1	Composition and size	$$\begin{array}{cc} \text {Mixed} \\ \text {untargeted} \end{array}$$	$$\begin{array}{l} \text {1) Scale GEGVs distribution to have } \mu = 0, sd = 1 \\ \text {2) Select hybrids whose scaled GEGVs are lower than } -\alpha \cdot sd \\ \text {and hybrids with scaled GEGVs higher than } \alpha \cdot sd \end{array}$$

$n_{set}$; the number of instances present in the set indicated in the subindex. For all matrices a subindex indicates that a subset is taken. For instance, $X_{TRS;All}$ represents the marker matrix whose rows are the individuals in the TRS and with all columns taken

*TRS* training set, *TS* test set, *i* an individual hybrid, *G* additive genomic relationship matrix, $\lambda$ shrinkage parameter, *X* can be the marker matrix or the markers can be replaced with principal components (for PCA_CDmean) or partial least squares variables (for PLS_CDmean), $diag({\cdot })$ main diagonal of a matrix, $mean({\cdot })$ average of all elements of a vector or matrix, *I* identity matrix, $argmax({\cdot })$ its argument has to be maximized, which was done using TrainSel heuristic, $\alpha$ parameter controlling TRS size in Tails_GEGVs_sd1

#### **Area under the curve**

We evaluated the effectiveness of an optimization method across different TRS sizes by quantifying its performance through the area under the curve (AUC) metric, as described by Fernández-González et al. [19]. Plotting the predictive ability against the TRS size (Fig. 4), AUC corresponds to the area under the curve that connects the available discrete values. We computed the AUC using Eq. 4.

4 $$\begin{aligned} \text {AUC} = \sum _{n = 1}^{nTRS-1} \left[ {\frac{\text {\textit{PA}}_{n} + \text {\textit{PA}}_{n+1}}{2}}\cdot (\text {\textit{TRS\_size}}_{n+1} - \text {\textit{TRS\_size}}_{n}) \right] \end{aligned}$$

Where *nTRS* represents the number of TRS sizes tested (four sizes, 20, 40, 60 and 80 % of the candidate set), PA represents the predictive ability and TRS_size represents the size of the TRS. To facilitate comparisons of AUC values across vastly different scenarios, they are expressed in relative terms as percentage gains relative to random sampling, as shown in the following equation:

5 $$\begin{aligned} AUC_{gain} = \left(\frac{AUC_{optimization}}{AUC_{random}} - 1\right) \times 100 \end{aligned}$$

### Supplementary Information

**Additional file 1.** Predictive ability of the gradient boosting machine model across all scenarios and repetitions for the training set (TRS) optimization methods tested using fixed values of the TRS size. The following columns are included: Method: TRS optimization method used; TRS_size: size of the TRS expressed as percentage of the candidate set size; Trait: phenotypic trait; Method_iter: iteration number for the optimization method; Model_iter: iteration number for the genomic selection model; Predictive_ability: correlation of predictions in test set and the empirical genotypic values; TS: year of the field trials used as test set; CS: years of the field trials used as candidate set (1 = one year prior to test set year, 2 = two years prior to test set year, etc.).

**Additional file 2.** Predictive ability of the gradient boosting machine model across all scenarios and repetitions for simultaneous optimization of training set (TRS) size and composition. The same columns as in Additional file 1 were used with the exception of "Method_size_optimization" (optimization method used to find optimal TRS size) and "Method_composition_optimization" (optimization method used to find optimal TRS composition).

**Additional file 3.** Additional analyses and results. Note 1, methodology used for calculating the genomic relationship matrices. Note 2, additional optimization methods tested in preliminary analyses and discarded due to poor performance. Note 3, methodology for heritability and variance components calculation. Note 4, additional details of the genomic selection models used. A comparison between the two models explained in the main text and a Bayesian B model with only additive effects fitted with BGLR R package [74] is also included. Note 5, detailed overview on the use of partial least squares for PLS_CDmean. Note 6, description of TrainSel hyperparameters used and detailed discussion about computational time of optimization. Note 7, guidelines for the interpretation of the Pareto front plots in multi-objective implementation and detailed analyses for each scenario not covered in the main text. After Note 7, several figures referenced in the main text were also included.

## Data Availability

The data analyzed in this study is not publicly available.
