# Correction: Maximizing efficiency in sunflower breeding through historical data optimization

**DOI:** 10.1186/s13007-024-01186-3

**Published:** 2024-05-09

**Authors:** Javier Fernández-González, Bertrand Haquin, Eliette Combes, Karine Bernard, Alix Allard, Julio Isidro y Sánchez

**Affiliations:** 1 Centro de Biotecnologia y Genómica de Plantas (CBGP, UPM-INIA)—Instituto Nacional de Investigación y Tecnologia Agraria y Alimentaria (INIA), Universidad Politécnica de Madrid (UPM), Campus de Montegancedo-UPM, Pozuelo de Alarcón, Madrid 28223 Spain; 2 Syngenta, Saint-Sauveur, France

**Correction: Plant Methods 2024 20:42** 10.1186/s13007-024-01151-0

"In the original publication of the article, it was brought to author’s attention that the first few rows of the table within Supplementary File 2 contained inaccuracies regarding the dating of the dataset. These inaccuracies do not affect the conclusions of our study but are crucial for the integrity and reproducibility of the research".

Correct ESM (Additional file 2) has been processed in this correction article.

The original article [1] has been corrected.

### Supplementary Information

Additional file 2.
