# Supplementary material for: Maximizing efficiency in sunflower breeding through historical data optimization
Source: Plant Methods. 2024 Mar 16;20:42. doi: 10.1186/s13007-024-01151-0 (PMC10943787; doi:10.1186/s13007-024-01151-0)
Supplement: Supplementary file 3 — Additional file 3. Additional analyses and results. Note 1, methodology used for calculating the genomic relationship matrices. Note 2, additional optimization methods tested in preliminary analyses and discarded due to poor performance. Note 3, methodology for heritability and variance components calculation. Note 4, additional details of the genomic selection models used. A comparison between the two models explained in the main text and a Bayesian B model with only additive effects fitted with BGLR R package [74] is also included. Note 5, detailed overview on the use of partial least squares for PLS_CDmean. Note 6, description of TrainSel hyperparameters used and detailed discussion about computational time of optimization. Note 7, guidelines for the interpretation of the Pareto front plots in multi-objective implementation and detailed analyses for each scenario not covered in the main text. After Note 7, several figures referenced in the main text were also included. [file 13007_2024_1151_MOESM3_ESM.pdf]

6 Supplementary Materials

6.1 Note 1. Calculation of the relationship matrices.

The calculation of additive and dominance relationship matrices was performed using "VanRaden" method in snpReady R package, version 0.9.6 [58]. The implementation performed by this package is shown in equations 6 and 7:

6.1.1 Additive Relationship Matrix

$$G = \frac{ZZ'}{tr(ZZ')/N} + 10^{-6}I \tag{6}$$

Where G is the additive relationship matrix, Z is the centered marker matrix in numeric form (please note that this is equivalent to the Z matrix in [64]), $tr(\cdot)$ indicates the trace of a matrix, N is the number of genotypes (rows) in the marker matrix and I is an identity matrix of the same dimensions as G. The addition of $10^{-6}I$ is a regularization step that ensures the non-singularity of G.

6.1.2 Dominance Relationship Matrix

$$D = \frac{WW'}{tr(WW')/N} \tag{7}$$

Where D is the dominance relationship matrix, N is the number of genotypes (rows) in the marker matrix, and W is a matrix containing the coefficients of the dominance deviation for each marker position in each genotype as in [65]. W can be computed from the marker matrix by replacing positions homozygous for the minor allele by $-2q^2$, positions homozygous for the major allele by $-2p^2$ and heterozygous positions by $2pq$; with p being the minor allele frequency and q the major allele frequency.

6.2 Note 2. Alternative methods for optimizing training set size and composition

The methods shown in Table S1 were not included in the main body of analyses due to their lackluster performance in preliminary testing:

- Tails_GEGVs_sd (untargeted): Approach based on standard deviation (see Table S1). The parameter α controls the TRS size and β controls if extreme outliers are allowed into the TRS. In this work we set $\alpha = 0.5, \beta = 2.5$ from previous experience.
- Tails_GEGVs_opt (untargeted): 1) sort GEGVs. 2) fit a sigmoidal and find the 2 inflexion points (Figure S10). 3) Take the extreme GEGVs for the TRS (GEGVs before the first inflexion point and after the second one).

Table S1: Summary of the training set optimization methods not included in the main analyses. All methods are based on Tails_GEGVs, which selects the n_{t1} hybrids with lowest GEGVs (lower tail) and the n_{t2} hybrids with highest GEGVs (higher tail). n_t; size of a unspecified tail (either the lower or the higher one). $0 < alpha < beta$; parameters in Tails_GEGVs_sd. p; number of markers. $m_{a,k}$; marker for hybrid a in locus k. $M^*_{TRS,k}$; markers in locus k for all hybrids in the training set (TRS). $count\_unique(\cdot)$; count the number of distinct, non missing values. $scale(\cdot)$; scale between 0 and 1 by subtracting lowest possible value and dividing by the difference between maximum and minimum possible values. $dist(\cdot)$; euclidean distance. $Geno\_freq_{set,k}(\cdot)$; relative frequency of the genotypes at locus k for the set indicated in the subindex, which can be the training set (TRS), the candidate set (CS) or the test set (TS). $Argmax(\cdot)$; maximize using grid search.

Method	Mechanism
Tails_GEGVs_sd	1) Scale GEGVs distribution to have $\mu = 0, sd = 1$<br>2) Select hybrids whose scaled GEGVs are between $-\alpha \cdot sd$ and $-\beta \cdot sd$ (lower tail) and hybrids with scaled GEGVs between $\alpha \cdot sd$ and $\beta \cdot sd$ (higher tail)
Tails_GEGVs_opt	1) Sort candidate set according to the GEGVs<br>2) Plot GEGVs against index number of all hybrids in the candidate set<br>3) Fit a sigmoidal function (Figure S10)<br>4) Find the two inflexion points of the sigmoidal<br>5) Select the hybrids before the first inflexion point for the TRS point and after the second one.
Tails_GEGVs_sym	1) Sort candidate set according to the GEGVs<br>2) Plot GEGVs against index number of all hybrids in the candidate set<br>3) Fit a sigmoidal function (Figure S10)<br>4) Find the two inflexion points of the sigmoidal<br>n_{t1} = Number of hybrids before the first inflexion point<br>n_{t2} = Number of hybrids after the second inflexion point<br>$n_3 = \frac{n_1+n_2}{2}$<br>5) Select n_3 top and n_3 bottom hybrids according to GEGVs
Tails_GEGVs_nuc	$Nuc\_div = \frac{\sum_{i=1}^{n_t}\sum_{j=(i+1)}^{n_t}\sum_{k=1}^{p}\left[\lvert m_{i,k}-m_{j,k}\rvert\right]}{[n_t(n_t-1)p]/2}$<br>TRS = Bottom tail + Top Tail<br>Bottom Tail:<br>1) Select n_{t1} hybrids with lowest GEGVs<br>2) Calculate Nuc_div for the n_{t1} hybrids selected<br>3) if Nuc_div reached plateau OR $n_{t1} \geq Max\_n_t$<br>stop<br>else<br>$n_{t1} = n_{t1} + d$<br>repeat from step 1<br>end if<br>Top Tail: same as for bottom tail but in step 1 we select n_{t2} hybrids with the highest GEGVs
OpTails	$Allelic\_div = \frac{\sum_{k=1}^{p} count\_unique(M^*_{TRS,k})}{p}$<br>$GEGVs\_low = n_{t1}$ lowest GEGVs; $GEGVs\_high = n_{t2}$ highest GEGVs<br>$OpTails = \frac{scale(Allelic\_div)}{1+\left[\frac{n_{t1}-n_{t2}}{n_{t1}+n_{t2}}\right]^2} - \frac{scale(var(GEGVs\_Low))+scale(var(GEGVs\_High))}{2}$<br>$Argmax(OpTails)$
OpTails_untarg	$Mean\_dist_{TRS,CS} = \frac{\sum_{k=1}^{p} dist(Geno\_freq_{TRS,k}, Geno\_freq_{CS,k})}{p}$<br>$GEGVs\_low = n_{t1}$ lowest GEGVs; $GEGVs\_high = n_{t2}$ highest GEGVs<br>$E1 = \frac{scale(min(GEGVs\_high))-scale(max(GEGVs\_low))}{2} - scale(Mean\_dist_{TRS,CS})$<br>$OpTails\_untarg = \frac{scale(E1)}{1+\left[\frac{n_{t1}-n_{t2}}{n_{t1}+n_{t2}}\right]^2}$<br>$Argmax(OpTails\_untarg)$
OpTails_targ	Similar to OpTails_untarg with a minor change in E1:<br>$E1 = \frac{scale(min(GEGVs\_high))-scale(max(GEGVs\_low))}{2} -$<br>$- scale(Mean\_dist_{TRS,CS} + Mean\_dist_{TRS,TS})$

- Tails_GEGVs_sym (untargeted): Same as Tails_GEGVs_opt but the number of individuals taken from both tails is the same (each tail has the average size of the two tails in Tails_GEGVs_opt).
- Tails_GEGVs_nuc (untargeted): Optimize using nucleotide diversity [72] following the idea that extreme individuals should be similar to each other (low nucleotide diversity) and average, uninteresting individuals should be more varied (high nulceotide diversity). Within each tail in Tails_GEGVs, we calculated nucleotide diversity for increasing tail sizes until it reaches a plateau. The optimal tail size is the point where the plateau is reached.
- OpTails (untargeted): Find a trade-off between minimizing variance of GEGVs within each tail and maximizing the allelic diversity (average number of different alleles across marker positions). A grid search was performed varying the size of both tails independently (Similar to the one shown in Figure S11), and maximizing the metric shown in Table S1
- OpTails_untarg (untargeted): Similar to OpTails but, instead of using allelic diversity, we calculated the average euclidean distance between genotypic frequencies in the TRS and the candidate set across marker positions. Furthermore, we relied on the maximum/minimum GEGV included in the lower/higher tail instead of GEGVs variances. Similarly to OpTails, we relied on a grid search (Figure S11) approach to maximize the metric shown in Table S1
- OpTails_targ (targeted): same as OpTails_untarg but in the calculation of *E1* (Table S1), instead of using the genotypic distance between TRS and candidate set, we sum the genotypic distance between the TRS and the candidate set and the genotypic distance between the TRS and the test set.

6.3 Note 3. Heritability calculation and variance extraction

Broad sense heritability was calculated using Cullis method [73] due to its robustness to unbalanced data. To that end, the first step model explained in the main body of the paper was fitted using all available phenotyped hybrids across all years and locations (32489 distinct genotypes). For the heritability within each year (needed for the multi-objective optimization), the same model was used replacing "year:location" effect by "location" effect and using only data from the corresponding year. These models allowed to extract the information needed for the calculation of heritability:

$$H^2_{Cullis} = 1 - \frac{\bar{v}^{BLUP}_{\Delta}}{2\sigma^2_g} \tag{8}$$

Where $\bar{v}^{BLUP}_{\Delta}$ is the average standard error of the genotypic BLUPs and σ^2_g is the genetic variance.

Variance extraction was performed on genotyped data only (16492 hybrids across all years and locations). The first step model was used to find the genotypic BLUPs and, subsequently, a GBLUP model was fitted. GCA and SCA variances were extracted from the latter and the additive to dominance variance ratio was calculated as the sum of the male and female GCA variances divided by the SCA variance.

6.4 Note 4. Additional details on Genomic Selection Models

6.4.1 BayesB Model

A BayesB model was also tested and compared to GBLUP and GBM explained in the main text. Equation 9 is the only model that accounts only for additive effects:

$$\boldsymbol{y} = \mathbf{1}\mu + X\boldsymbol{g} + \boldsymbol{\varepsilon}, \tag{9}$$

where $\boldsymbol{y}$ is a vector of BLUPs for hybrids, $\mathbf{1}$ is a vector of ones μ is the overall mean, X is the hybrid marker matrix, $\boldsymbol{g}$ is a vector of marker effects and $\boldsymbol{\varepsilon}$ is a vector of residuals. This model was fitted under a bayesian framework using BGLR package [74] assuming the following prior density [8]:

$$\sigma^2_{gi} = 0 \quad \text{with probability } \pi, \tag{10}$$

$$\sigma^2_{gi} \sim \chi^{-2}(v,S) \quad \text{with probability } 1-\pi, \tag{11}$$

Where σ^2_{gi} refers to de variance of the marker effect i and $\chi^{-2}(v,S)$ is an inverted chi-square distribution with $v = 4.234$ and $S = 0.0429$. For more details, see [8]

6.4.2 Comparison of models. Methodology

Three GS statistical models were compared: BayesB (only additive effects), GBLUP and GBM (additive and non-additive effects, explained in the main text). Table S2 contains the different combinations of years in the TS and TRS in which the models were tested. The combinations chosen included evaluation of models when TRS and TS data belonged to the same year, recent TRS just one year prior to the TS and older TRS data. For the latter case, the most realistic scenario is having data available from the oldest data to the most recent one, and it would not make sense to exclude the recent data when it is available. Therefore, for evaluating model performance in the presence of old data, we assumed that all available years prior to the TS were included into the TRS. Intermediate scenarios in which not all older years are included

in the TRS would be possible, but we decided not to study them due to the extremely high computational cost of fitting all models in additional scenarios.

Table S2: Year combinations for training and test sets, utilized for genomic selection model comparisons. *Scenario 7 employed a 4-fold cross-validation exclusive to Year 7 data. In this approach, Year 7 data is randomly split into separate training and test sets. This ensures exclusive use of Year 7 data without overlap between training and testing sets, facilitating a fair evaluation.

	Scenario 1	Scenario 2	Scenario 3	Scenario 4	Scenario 5	Scenario 6	Scenario 7
Training set years	4	1-4	5	1-5	6	1-6	7*
Test set years	5	5	6	6	7	7	7*

The relative performance of the three GS models tested in the scenarios described in Table S2 is summarized in Figures S1 - S3

6.4.3 Comparison of Models. Results

We found that all models exhibited a substantially superior predictive ability when making predictions within year 7, as opposed to predictions across years, highlighting the significant impact of year effects on genomic selection performance, as also noted in many previous studies [44, 46]. Among the tested models, BayesB had the worst performance, while GBLUP consistently performed better than BayesB, but was still substantially inferior to GBM, particularly in YLD. We also assessed the computational time required by each model, which revealed that GBLUP was the slowest method, followed by BayesB, while GBM was the fastest.

While the ranking of the models was consistent across all traits, the relative difference in their performance varied considerably. For instance, in YLD, the predictive ability of GBM and GBLUP was on average 17.61% and 9.46% higher than that of BayesB respectively (Figure S1). These values dropped to 6.81% and 4.22% in GM (Figure S2) and 4.62% and 3.33% in OIL (Figure S3). We selected GBM as the primary model for evaluating TRS optimization methods in all traits and scenarios based on these results.

The likely reason behind BayesB subpar predictive ability is its consideration of only additive effects, which is not ideal for hybrid breeding scenarios where dominance effects play a crucial role [57, 75, 76]. Both GBLUP and GBM models considered dominance effects, with GBM being able to implicitly consider other effects such as epistasis. Although GBM's advantage over GBLUP was minor in some traits (GM and OIL), it consistently performed better in the low heritability trait YLD (Table 4). Yield was also the trait with the highest

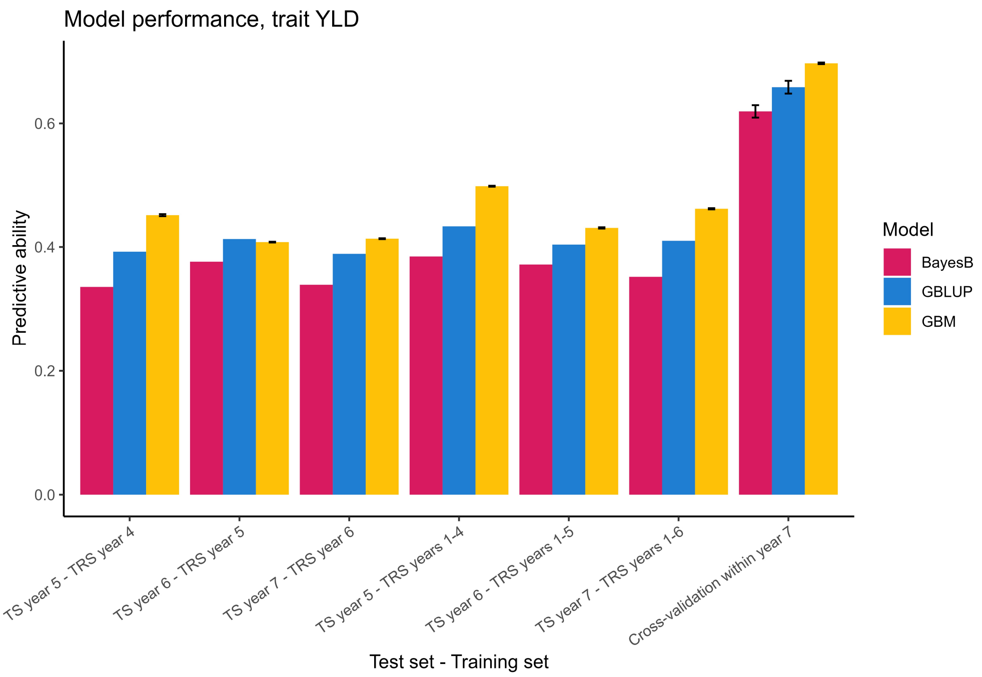

Figure S1: Barplot comparing the predictive abilities of different models used for the grain yield (YLD) trait, across seven different combinations of test and training years. The error bars represent the standard error of the mean for the 30 repetitions performed in the gradient boosting machine, and the four folds of the cross-validation performed within the year 7.

importance of dominance relative to additive effects (Table 4). As such, it is possible that GBM is better able to capture dominance effects than the GBLUP (GCA+SCA) model, which is particularly important for low heritability traits [75]. Nevertheless, GBM had a disadvantage in its random start, which can potentially impair its consistency. The minimal error bars in Figure S1 indicate that this effect is negligible.

6.4.4 Gradient Boosting Machine hyperparameter tuning

Hyperparameter tuning was performed using a sequential grid search where different hyperparameter values were evaluated using a 5-fold cross-validation (using xgb.cv() function and selecting hyperparameters that minimized root mean squared error in the test fraction of the cross-validation). As there were too many hyperparameters to test all possible combinations between them, the most important ones were tuned first, their optimal values were fixed for subsequent tests and then the next hyperparameters were tuned:

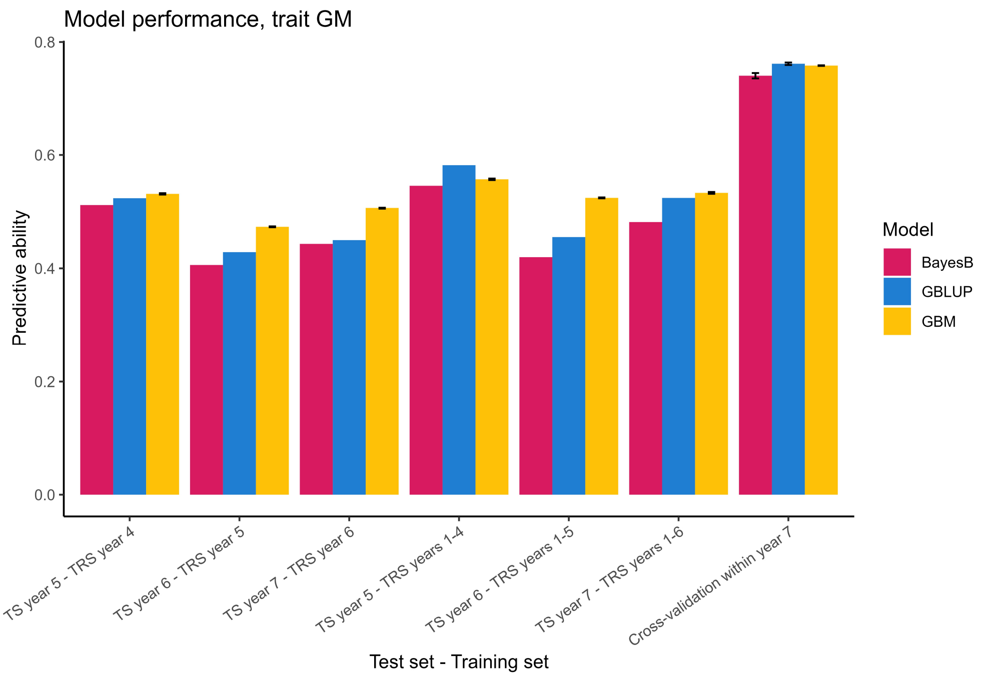

Figure S2: Barplot comparing the predictive abilities of different models used for grain moisture (GM), across seven different combinations of test and training years. The error bars represent the standard error of the mean for the 30 repetitions performed in the gradient boosting machine, and the four folds of the cross-validation performed within the year 7.

1. Fix initial parameters: eta = 0.2; max_depth = 5; min_child_weight = 1; gamma = 0; subsample = 0.8; colsample_bytree = 0.8. It is important to note that the initial learning rate (eta) is high to accelerate model convergence.
2. With the initial parameter fixed, tune the number of boosting rounds between 50 and 1000, considering that the optimal has been reached if there is no improvement after 50 consecutive rounds.
3. Using the optimal number of rounds, tune different combinations of max_depth (between 3 and 7, with a precision of ± 1) and min_child_weight (between 2 and 6, with a precision of ± 1).
4. gamma was tuned testing values between 0 and 0.40, with a precision of ± 0.05.
5. Re-calibrate number of boosting rounds for the tuned parameters.
6. Different combinations of subsample (between 0.70 and 0.90, with a precision of ± 0.05) and colsample_bytree (between 0.70 and 0.90, with a precision of ± 0.05) were tested.
7. Tune regularization parameter reg_alpha (between 10^{-6} and 10^{2}).

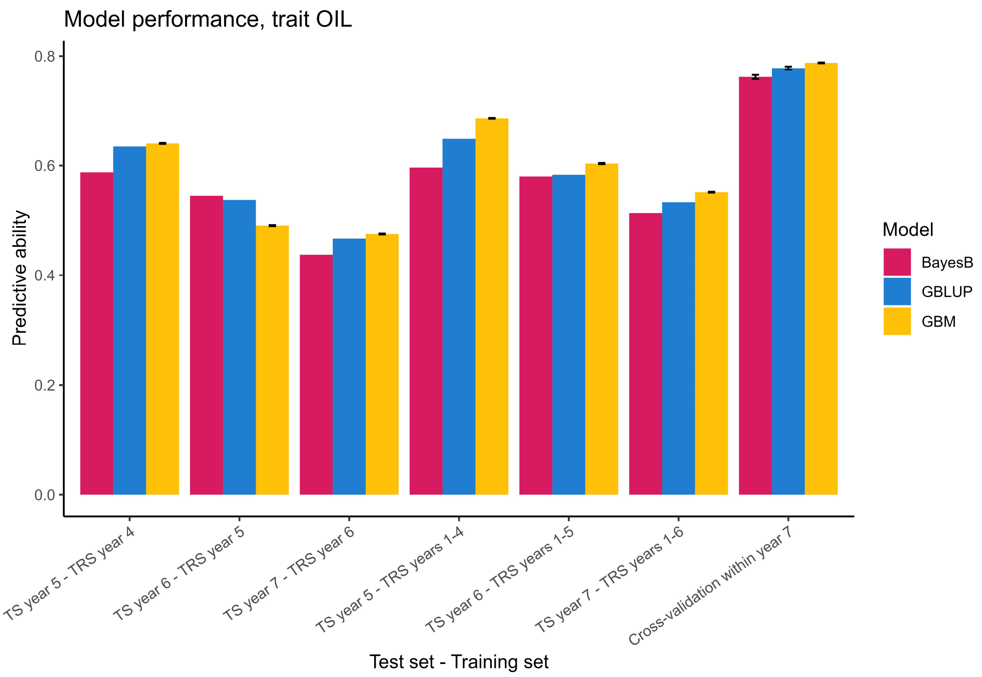

Figure S3: Barplot comparing the predictive abilities of different models used for percentage of oil (OIL), across seven different combinations of test and training years. The error bars represent the standard error of the mean for the 30 repetitions performed in the gradient boosting machine, and the four folds of the cross-validation performed within the year 7.

8. Reduce learning rate and simultaneously tune number of boosting rounds. Learning rate (eta) values between 0.01 and 0.2 with a precision of ± 0.025 were tested and number of rounds between 50 and 1000 were considered.

6.5 Note 5. Partial least squares for dimensionality reduction

In PLS_CDmean, we used partial least squares (PLS) to reduce dimensionality in a similar way in which we used principal component analysis (PCA) in PCA_CDmean. However, in contrast to PCA, PLS requires both genotypic information and a genotypic value for the trait of interest, which is not avaliable for the TS *a priori*, which would make targeted PLS_CDmean impossible. To solve this issue, we performed 3 steps: i) use GBM model trained on the candidate set to calculate the GEGVs in the candidate set and TS, ii) compute PLS maximizing covariance between the GEGVs (acting as labels) and the marker data (acting as predictor variables), iii) use the first 200 PLS-generated variables in CDmean in a similar way as the 200 first PCs were used in PCA_CDmean (Table 6). PLS generates latent variables based on the covariance between labels (GEGVs) and predictor variables (markers), while PCA is based

solely on the variance of the markers. That way, dimensionality reduction can be achieved in a similar way to PCA with the added advantage that the genotypic and phenotypic information can be considered simultaneously. The main disadvantage of this approach is that it requires a GS model to be trained on the entire candidate set to be able to predict the labels for the TS.

6.6 Note 6. TrainSel parameters and computational time

There are two factors that can make computational time problematic for an optimization method:

- Costly matrix multiplications or inversions. For the sake of simplicity, we will assume cubic time complexity for both.
- Reliance on a search heuristic that needs to compute the evaluation metric repeatedly. In this work we used the TrainSel heuristic, version 2.0 [69] with 1000 iterations, population size of 200, 10 simulated annealing steps per iteration and 5 elite solutions kept per iteration. With these parameters, the evaluation metric had to be computed $200 + (200 - 5) * 1000 + 5 * 10 * 1000 = 245,200$ times every time optimization was performed, unless convergence was reached sooner.

Avg_GRM, Min_GRM, Tails and Random sampling had a negligible computational time, as they did not require costly matrix operations nor did they rely on a search heuristic.

Tails_GEGVs and Tails_GEGVs_sd1 were extremely fast once the GEGVs were available. However, the GEGVs calculation requires training a GS model on the candidate set, which can be computationally intensive. In this work, we used a GBLUP model as implemented in Sommer [66], which has a cubic time complexity, $O(n^3)$, where n is the number of hybrids.

Avg_GRM_self and Avg_GRM_MinMax both have quadratic time complexity ($O(n^2)$). They don't require any matrix operation, but both involve subsetting an extremely large relationship matrix. This can be done very efficiently in Fortran if the matrix is stored as a one-dimensional array. Further time can be saved if the symmetry of the relationship matrix is exploited, only accessing the upper triangular part. As a result, both evaluation metrics were extremely fast and optimization could be performed in a few hours despite the reliance on TrainSel.

The **CDmean** implementation used here is limited by $O(n^2p)$ matrix operations if $n >> p$ or by $O(np^2)$ operations if $p >> n$, where n is the number of hybrids and p is the number of markers. When dimensionality reduction (PCA or PLS) is used, p is the number of PCs or PLS-derived variables considered (200 in this work), which makes $n >> p$. Therefore, we can approximate the time complexity of both PCA_CDmean and PLS_CDmean as $O(n^2p)$, which was viable despite the need for a search heuristic.This implementation was vastly faster than

classical CDmean [21], which has a $O(n^3)$ time complexity and would not be viable with the large datasets involved in this work.

Multi-objective optimization relies on a search heuristic and it requires three criteria to be calculated per iteration, which can make it seem computationally inefficient at first glance. However, the maximization of diversity and relatedness to the test set is very fast (similar to Avg_GRM_MinMax, $O(n^2)$, no computationally intensive matrix operations), while the average heritability is trivial to compute (it is just the average of the heritability of each year included in the training set). Furthermore, the first step models needed for heritability calculation converge fast, as no large genomic relationship matrix is used in them and lme4 package [63] implementation is extremely efficient. Finally, while multi-objective optimization uses the TrainSel heuristic, convergence is reached very fast, massively reducing computational burden. In other optimization methods, the candidate set were thousands of genotypes and numerous combinations of them needed to be tested to find the optimal subset that would conform the TRS. Therefore, the search space was extremely large and a lot of TrainSel iterations were needed. In contrast, In multi-objective optimization, the candidate set comprises just a handful of years, hugely reducing the search space and making TrainSel converge extremely fast. As a result, multi-objective optimization can be easily run in just a few minutes at worst for any realistic number of years in the candidate set.

6.7 Note 7. Interpretation of results of multi-objective optimization to select years in training set

As multi-objective optimization outputs a series of potentially good solutions, it is important to be able to find the best one among them. Here, we present the guidelines found to generate the best results and provide the interpretation for all traits and years in the test.

The guidelines we have found to select the best combination of year are the following:

- Diversity is the most important variable. If there is a solution that clearly has more diversity than the rest it should be selected. If there are several solutions with negligible differences in diversity among each other, the other variables should be considered.
- Among solutions with similar diversity, if there is one with substantially higher heritability and/or relationship to the TS than the rest, it should be selected.
- The number of years that the solution includes must also be considered, as more years result in a better estimation of year effects and removal of environmental effects in the first step modelling. If one solution only has slightly better heritability and relationship to the TS than others with similar diversity but it includes less years, the solution with more years should be selected.
- All things being equal, prioritize solutions with the most recent years.

Percentage of oil, Test set year 6

Step 1: Find the year combinations with highest diversity (Figure S4 A,B). Combinations *a, h and f* are the best. The rest are discarded.

Step 2: Among combinations *a, h and f*, find the one with highest heritability, relationship to the TS and number of years (Figure S4 A,B,C,D). Solutions *f and h* have slightly higher heritability and relationship to the TS than solution *a*, but they include only 3 years, while solution *a* has 4 (a 33% increase in training set years). Therefore, solution *a* is selected.

In Table 1, solution *a* is found to have the best predictive ability for GBM (0.557) and one of the highest in GBLUP (0.546). It is important to note that the inverse happens for solution *h*, which almost has the best predictive ability in GLBUP (0.555) but lags slightly behind in GBM (0.549).

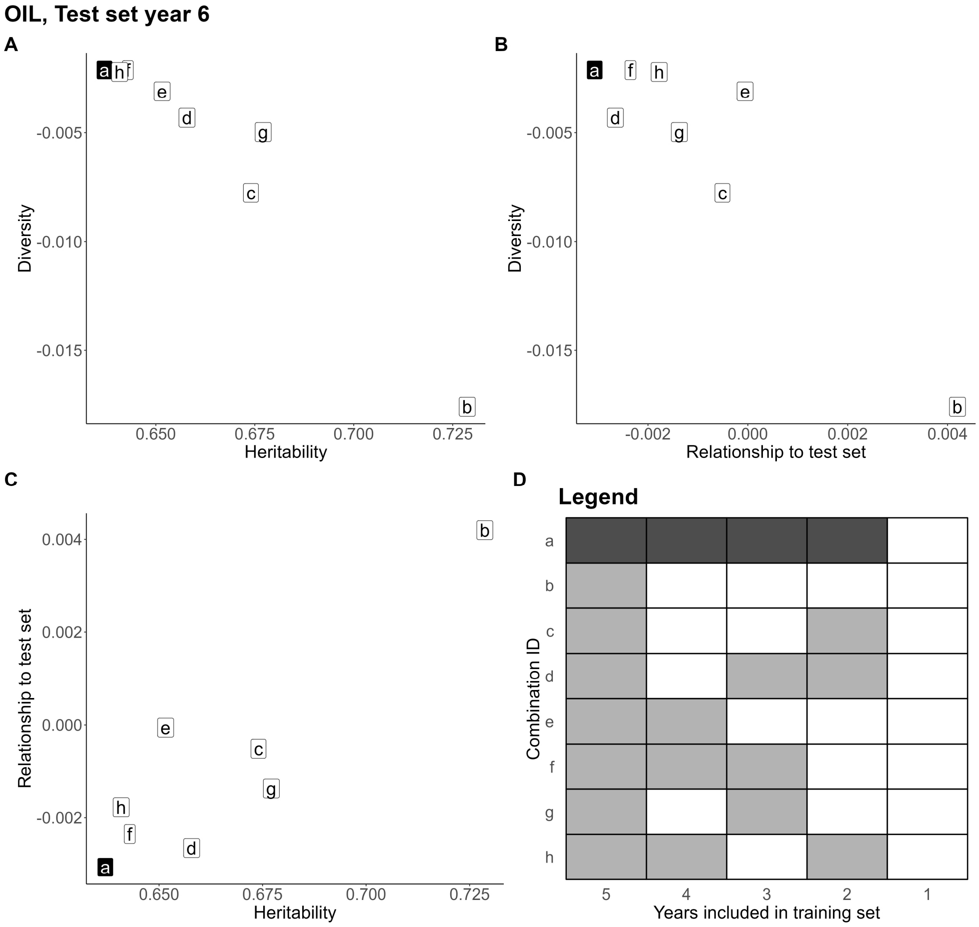

Figure S4: Results of the multi-objective optimization that maximized diversity, relationship to the TS and average heritability in the percentage of oil trait when the TS was year 6. The solutions found by the optimization algorithm conform a three-dimensional Pareto front. To facilitate result visualization, the results have been displayed in 3 two-dimensional plots that show all pairwise combinations between the variables maximized in the optimization: A) Diversity against heritability. B) Diversity against relationship to the TS. C) Relationship to the TS against heritability. Each letter in these plots corresponds to a combination of years in the Pareto front and its composition can be found in plot D), where a gray square means that the year has been included in the training set. We have highlighted a year combination with a darker color to indicate that it corresponds to the best solution.

Percentage of oil, Test set year 7

Step 1: Find the year combinations with highest diversity (Figure S5 A,B). Combinations *i and d* are the best. The rest are discarded.

Step 2: Among combinations *i and d*, find the one with highest heritability, relationship to the TS and number of years (Figure S5 A,B,C,D). Solution *d* has slightly higher heritability and relationship to the TS than solution *i*, but it includes only 4 years while solution *i* has 5 (a 25% increase in training set years). Therefore, solution *i* is selected.

In Table 1, solution *i* is found to have extremely high predictive abilities. For GBM, its predictive ability is 0.470, only outperformed by selecting all years (0.478) and all years except year 3 (0.471). For GBLUP, solution *i* reaches a predictive ability of 0.493, only behind selecting all years except 1 and 3 (0.495). It is important to note that the year combinations that outperform solution *i* are different for GBM and GBLUP, highlighting the difficulty of finding a universally optimal solution. However, multi-objective optimization is able to select a solution with close to optimal performance in all scenarios.

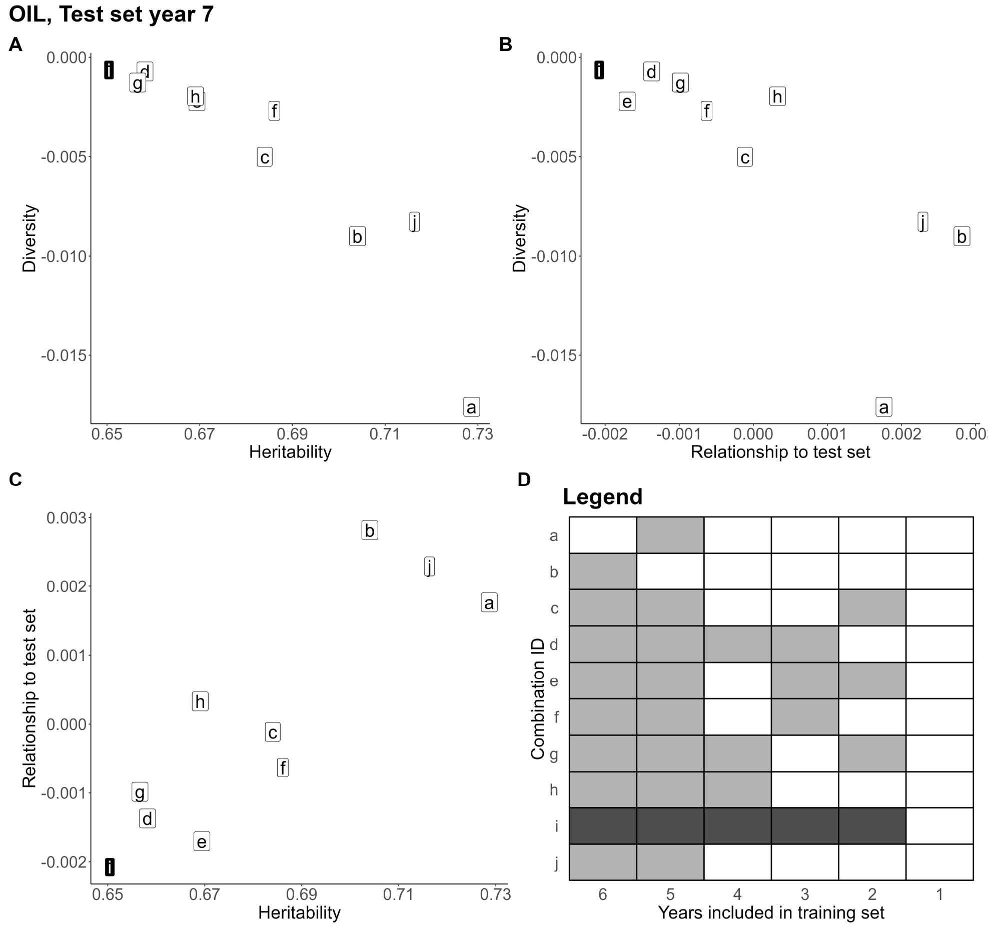

Figure S5: Results of the multi-objective optimization that maximized diversity, relationship to the test set and average heritability in the percentage of oil trait when the test set was year 7. The solutions found by the optimization algorithm conform a three-dimensional Pareto front. To facilitate result visualization, the results have been displayed in 3 two-dimensional plots that show all pairwise combinations between the variables maximized in the optimization: A) Diversity against heritability. B) Diversity against relationship to the test set. C) Relationship to the test set against heritability. Each letter in these plots corresponds to a combination of years in the Pareto front and its composition can be found in plot D), where a gray square means that the year has been included in the training set. We have highlighted a year combination with a darker color to indicate that it corresponds to the best solution.

Grain Moisture, Test set year 6

Step 1: Find the year combinations with highest diversity (Figure S6 A,B). Combinations *a, b and d* are the best. The rest are discarded.

Step 2: Among combinations *a, b and d*, find the one with highest heritability, relationship to the TS and number of years (Figure S6 A,B,C,D). Solution *d* is the one with highest heritability and relationship to the TS, but not by a large margin. In contrast, solution *b* includes 4 years (33% more than the 3 years in solution *d*). Therefore, solution *b* is selected.

In Table 1, solution *b* is found to have the second best predictive ability for both models (0.463 for GBM and 0.425 for GBLUP), only slightly lower than using all years in the training set. Multi-objective optimization has found a very good but suboptimal solution in this case. This may be due to an environmental similarity between the unselected year 1 and the TS year.

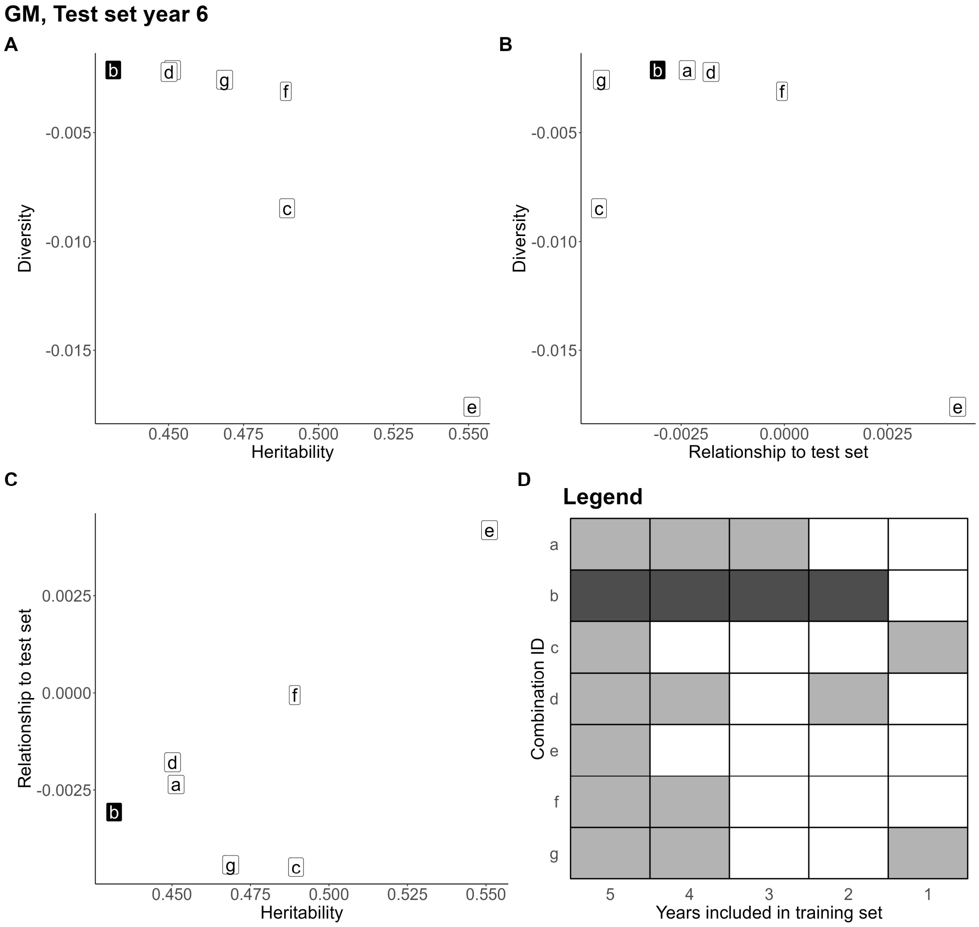

Figure S6: Results of the multi-objective optimization that maximized diversity, relationship to the test set and average heritability in grain moisture trait when the test set was year 6. The solutions found by the optimization algorithm conform a three-dimensional Pareto front. To facilitate result visualization, the results have been displayed in 3 two-dimensional plots that show all pairwise combinations between the variables maximized in the optimization: A) Diversity against heritability. B) Diversity against relationship to the test set. C) Relationship to the test set against heritability. Each letter in these plots corresponds to a combination of years in the Pareto front and its composition can be found in plot D), where a gray square means that the year has been included in the training set. We have highlighted a year combination with a darker color to indicate that it corresponds to the best solution.

Grain Moisture, Test set year 7

Step 1: Find the year combinations with highest diversity (Figure S7 A,B). Combinations *c, a and j* are the best. The rest are discarded.

Step 2: Among combinations *c, a and j*, find the one with highest heritability, relationship to the TS and number of years (Figure S7 A,B,C,D). Solution *j* is the one with highest heritability and relationship to the TS, but not by a large margin. In contrast, solution *c* includes 5 years (25% more than the 4 years in solution *j*). Therefore, solution *c* is selected.

In Table 1, solution *c* is found to have a very high predictive ability of 0.475 for GBM and 0.480 for GBLUP. This is sligtly higher than solution *j* for GBM and sligtly lower for GBLUP. Both solutions *c and j* are also slightly worse than selecting all years for both models. Multi-objective optimization has found a very good but suboptimal solution in this case. This may be due to an environmental similarity between the unselected year 1 and the TS year.

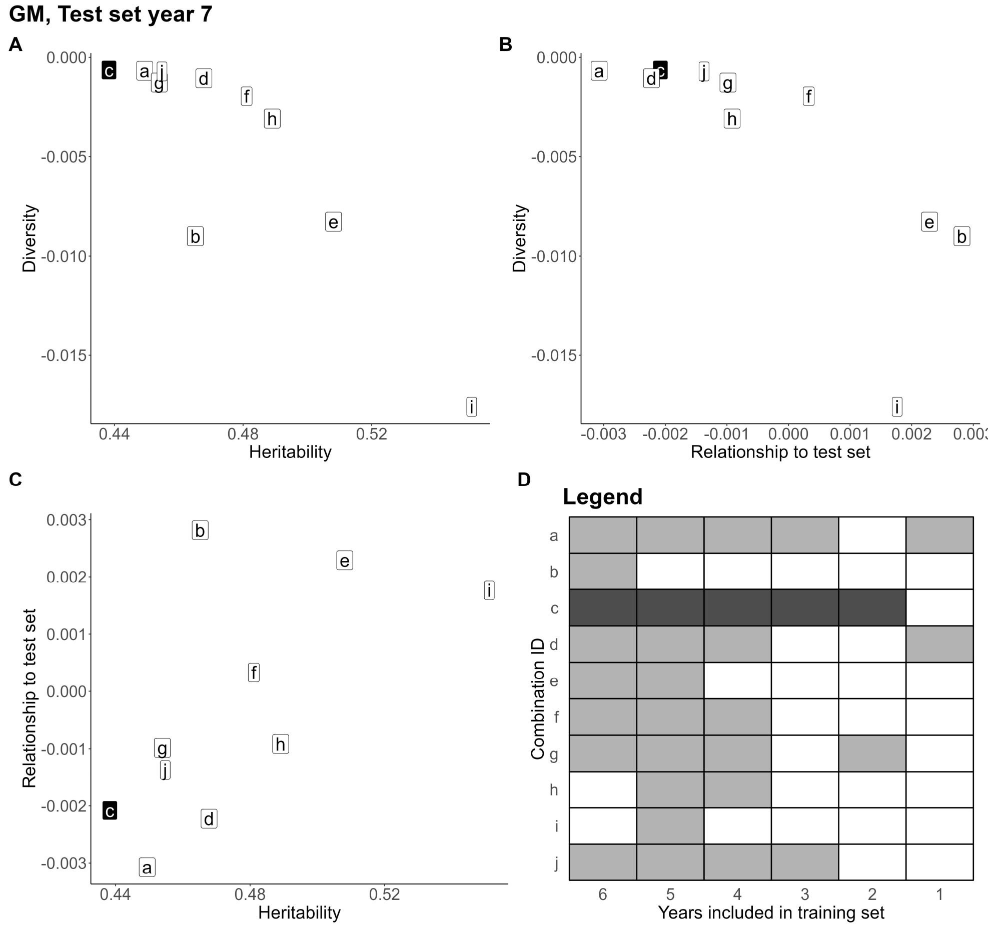

Figure S7: Results of the multi-objective optimization that maximized diversity, relationship to the test set and average heritability in grain moisture trait when the test set was year 7. The solutions found by the optimization algorithm conform a three-dimensional Pareto front. To facilitate result visualization, the results have been displayed in 3 two-dimensional plots that show all pairwise combinations between the variables maximized in the optimization: A) Diversity against heritability. B) Diversity against relationship to the test set. C) Relationship to the test set against heritability. Each letter in these plots corresponds to a combination of years in the Pareto front and its composition can be found in plot D), where a gray square means that the year has been included in the training set. We have highlighted a year combination with a darker color to indicate that it corresponds to the best solution.

Yield, Test set year 7

Step 1: Find the year combinations with highest diversity (Figure S8 A,B). Combinations *d, j and b* are the best, with combinations *e and i* being extremely close. The rest are discarded.

Step 2: Among combinations *d, j and b*, it is not clear which one is better for heritability and relationship to the TS. Solution *d* is discarded as it only comprises 4 years while *j and b* have 5. Years in solution *j* are more recent than the ones in solution *b* and, as a result, the latter is discarded. Solution *e* has slightly lower diversity than *j* but it does not present higher heritability or relationship to the TS and is discarded. Solution *i* has much higher heritability and relationship to the TS than solution *j* at the expense of slightly lower diversity and including one less year. It is not clear whether solution *j or i* is better, but solution *j* is selected as diversity is the most important variable.

In Table 1, both solutions *i and j* present extremely similar predictive abilities for both models and outperform any other solution.

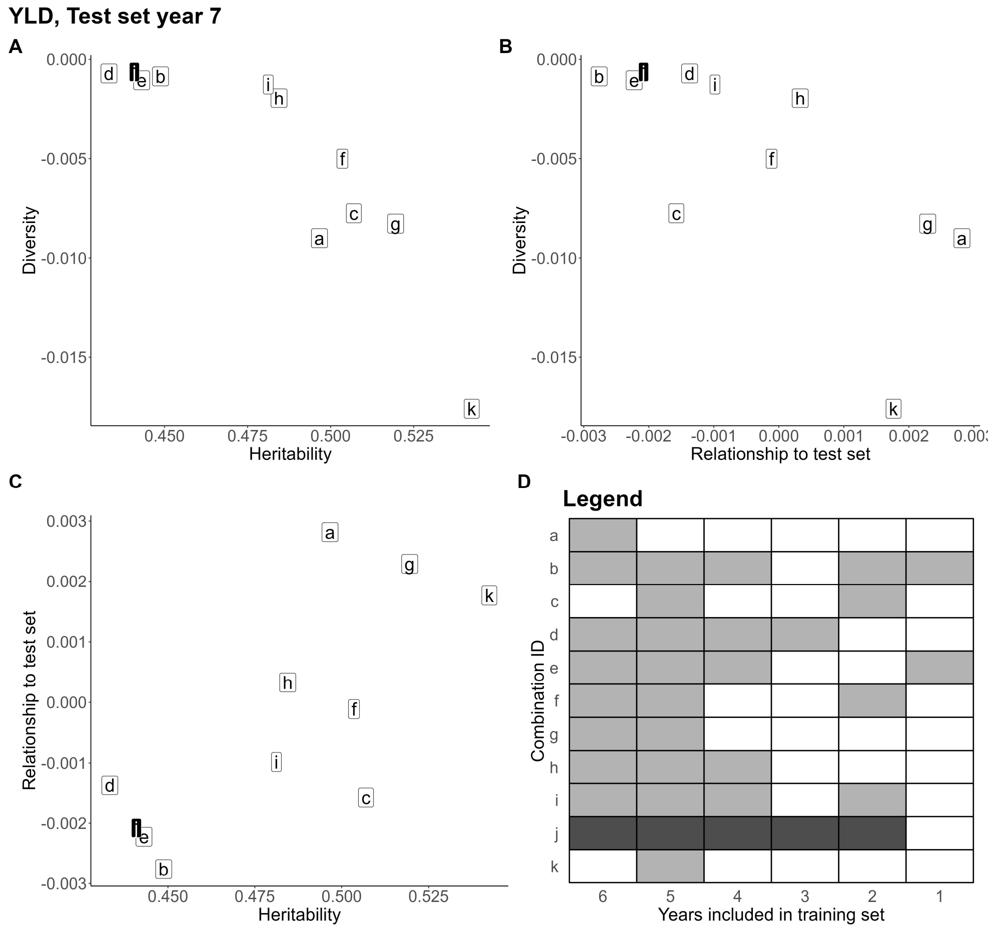

Figure S8: Results of the multi-objective optimization that maximized diversity, relationship to the test set and average heritability in yield trait when the test set was year 7. The solutions found by the optimization algorithm conform a three-dimensional Pareto front. To facilitate result visualization, the results have been displayed in 3 two-dimensional plots that show all pairwise combinations between the variables maximized in the optimization: A) Diversity against heritability. B) Diversity against relationship to the test set. C) Relationship to the test set against heritability. Each letter in these plots corresponds to a combination of years in the Pareto front and its composition can be found in plot D), where a gray square means that the year has been included in the training set. We have highlighted a year combination with a darker color to indicate that it corresponds to the best solution.

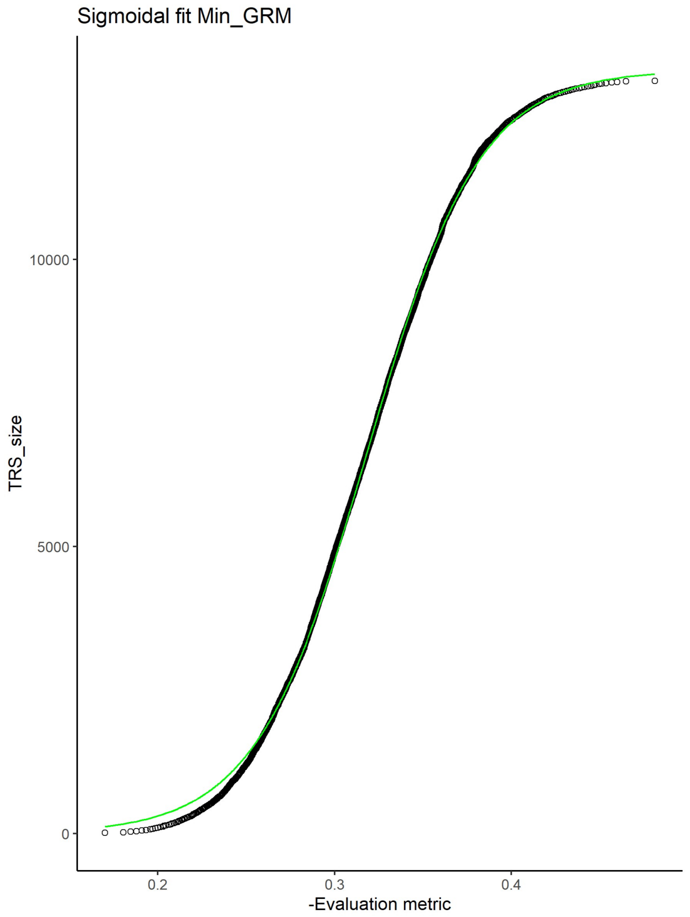

Figure S9: Evolution of the value of the Min_GRM evaluation metric as the training set size increases. The green line corresponds to the best sigmoidal fit to the empirical values. This plot corresponds to the scenario in which the test set is the year 7 and the candidate set comprises years 1-6. Min_GRM has solely been utilized in literature to optimize the training set composition, however, we applied it here to optimize the size of the training set as well. To accomplish this, we tested all sizes ranging from 0 to 100% of the candidate set, and Min_GRM optimization was performed for each. Plotting the training set size against the opossite of the Min_GRM evaluation metric yields a distinct sigmoidal shape. A steep slope in this plot means that the training set size can be expanded with a minimal decrease in the evaluation metric. Consequently, an optimal training set size has to contain all individuals situated in the central section of the sigmoidal curve (with a high slope). As such, the second inflexion point of the sigmoidal curve, which can be analytically determined using the second derivative of the sigmoidal function fitted to the data, coincides with the optimal training set size. Once the optimal size is determined, the training set composition can be optimized using Min_GRM or any other method.

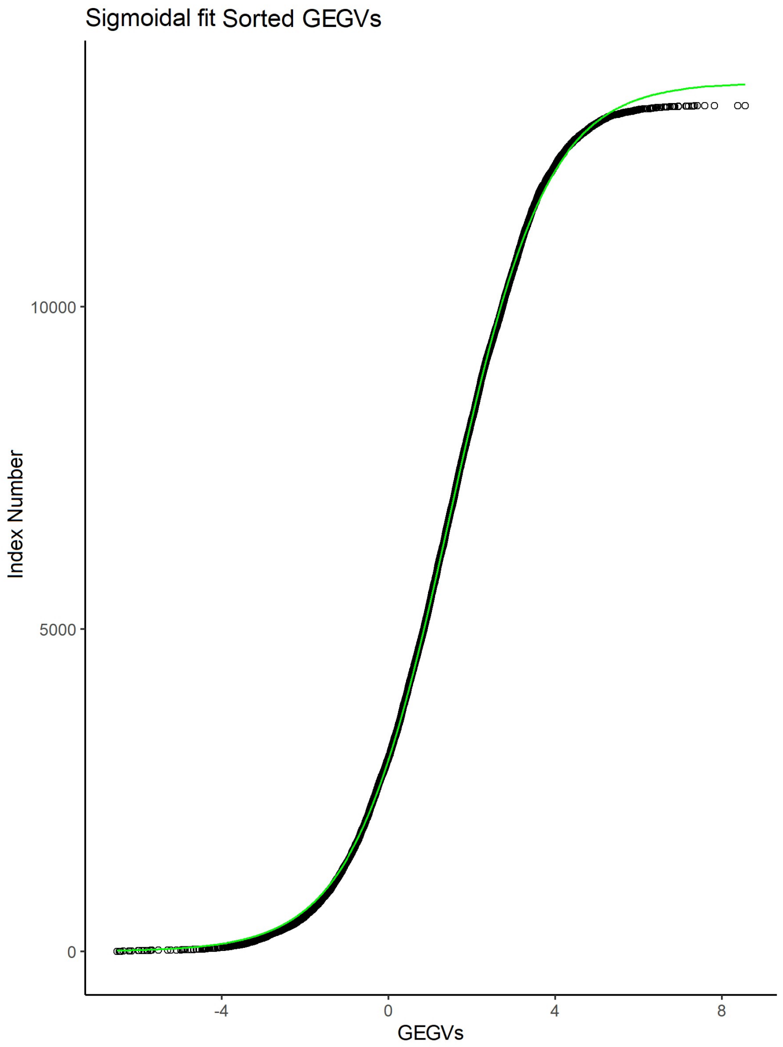

Figure S10: Distribution of the genomic estimated genotypic values (GEGVs) for grain yield sorted from lower to higher within the candidate set comprising years 1-6. The green line corresponds to the best sigmoidal fit to the empirical values.

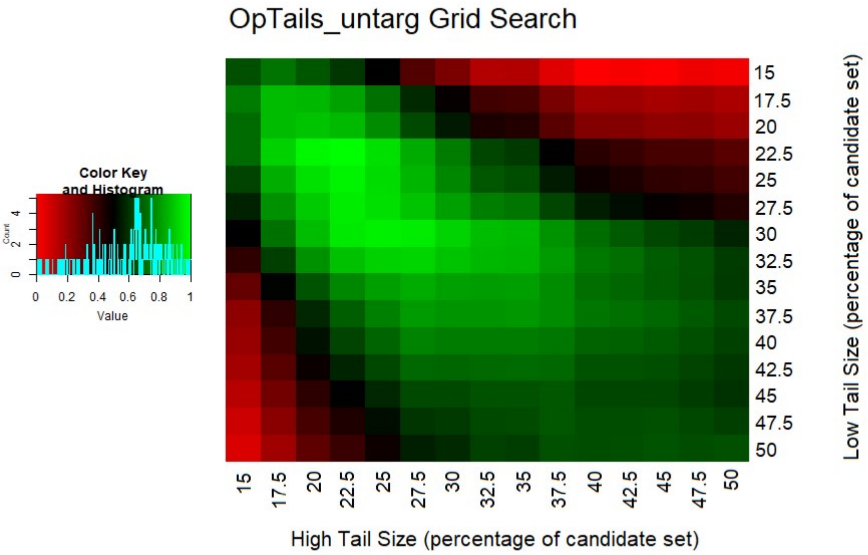

Figure S11: Grid search using OpTails_untarg optimization method for varying sizes of the lower and higher tails. The optimal training set size corresponds to the tile with the highest value of OpTails_untarg evaluation metric. This plot corresponds to the scenario in which the test set is the year 7 and the candidate set is the year 6

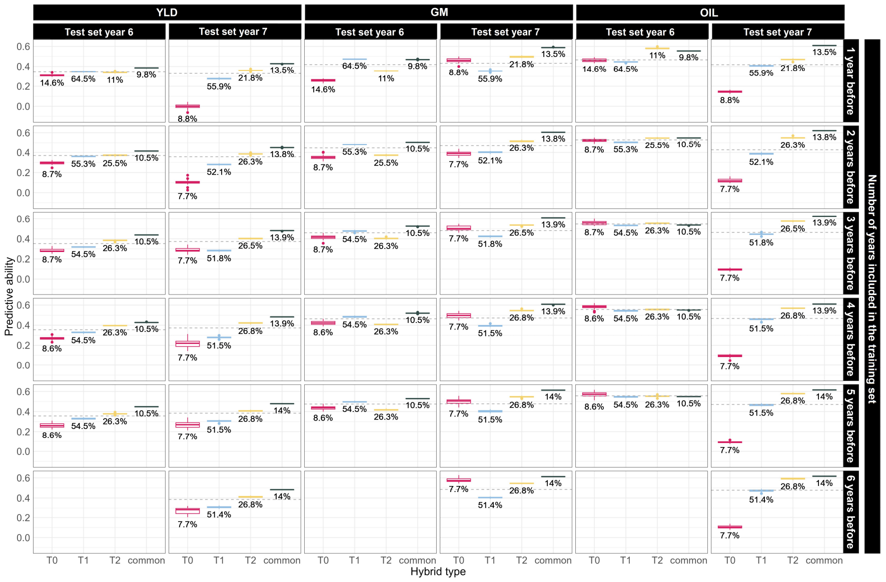

Figure S12: Boxplot of predictive abilities across modelling iterations. The different plots in the grid correspond to combinations of traits and test set years (top) and training sets (right). The training set numbers in the headers at the right correspond to the number of consecutive years preceding the test set data that were included in the training population. The training sets were built using all data from the corresponding years without performing training set optimization. Within each plot, the hybrids of the test set have been split in four different groups: T0; no parents in common with any hybrid in the training set. T1; one parent in common with at least one hybrid belonging to the training set. T2; both parents of the hybrid were also parents of hybrids of the training set. Common; the same hybrid combination was found in both the training and test sets. The dashed horizontal line is the average predictive ability of all hybrids within the scenario. The percentage below each box refers to the proportion of the total test set comprised by the corresponding hybrid type.

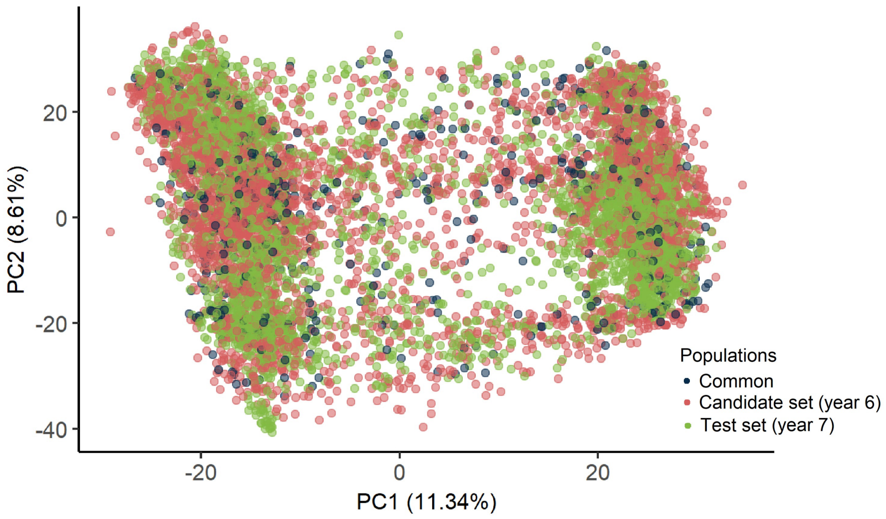

Figure S13: Plot of the first two principal components calculated on genome-wide marker data. The percentage next to the name of each principal component indicates the percentage of genetic variance it explains. Each solid circle represents a hybrid belonging to the test set (year 7, green), the training set (year 6, red) of both (blue). The aim of this plot is showing that the training and candidate set almost fully overlap in the genetic space. In this case, data from years 6 and 7 were used as an example, but similar results were obtained in all scenarios tested.

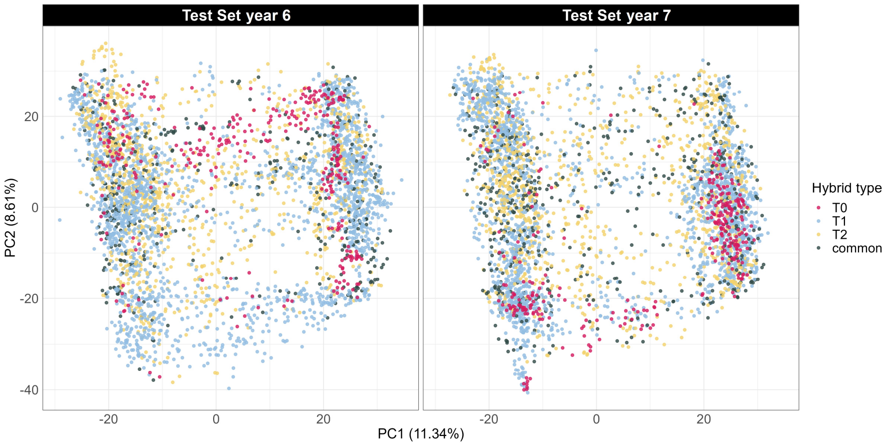

Figure S14: Plot of the first two principal components calculated on genome-wide marker data. The percentage next to the name of each principal component indicates the percentage of genetic variance it explains. Each solid circle represents a hybrid belonging to a test set comprised of all data from year 6 (left) or year 7 (right). The colors indicate a qualitative relationship between each hybrid of the test set and a training set comprised of all older data available: T0; hybrids of the test set have no parents in common with any hybrid in the training set. T1; one parent in common with at least one hybrid belonging to the training set. T2; both parents of the hybrid were also parents of hybrids of the training set. Common; the same hybrid combination was found in both the training and test sets.
